# Supplementary material for: A systematic review and meta-analysis of yoga for arterial hypertension
Source: PLoS One. 2025 May 14;20(5):e0323268. doi: 10.1371/journal.pone.0323268 (PMC12077774; doi:10.1371/journal.pone.0323268)
Supplement: S2 Table — (DOCX) [file pone.0323268.s002.docx]

**S2 Table: Subgroup analyses of yoga vs. waitlist control**

| **Subgroup or outcome** | **No. of studies** | **No. of patients (yoga)** | **No. of patients (usual care)** | **Mean difference (95% confidence interval)** | **P (overall effect)** | **Heterogeneity**  **I^2^; Chi^2^; Tau^2^; P** |
| --- | --- | --- | --- | --- | --- | --- |
| **Participant group** |  |  |  |  |  |  |
| Prehypertension |  |  |  |  |  |  |
| Systolic blood pressure | 3 | 219 | 221 | -3.79 (-6.36; -1.22) | <0.01 | 84%; 12.46; 4.22; <0.01 |
| Diastolic blood pressure | 3 | 219 | 221 | -2.40 (-4.13; -0.67) | <0.01 | 85%; 13.03; 1.92; <0.01 |
| Heart rate | 2 | 168 | 170 | -4.80 (-9.70; 0.11) | 0.06 | 88%; 8.42; 11.19; <0.01 |
| Prehypertension and  hypertension (mixed) | | | | | | |
| Systolic blood pressure | 4 | 136 | 141 | -8.67 (-18.92; 1.58) | 0.10 | 95%; 57.17; 102.70; <0.01 |
| Diastolic blood pressure | 4 | 136 | 141 | -4.78 (-9.23; -0.34) | 0.04 | 93%; 45.07; 18.81; <0.01 |
| Heart rate | 2 | 56 | 61 | -0.39 (-4.72; 3.94) | 0.86 | 55%; 2.23; 6.63; 0.14 |
| Hypertension | | | | | | |
| Systolic blood pressure | 19 | 685 | 605 | -8.71 (-11.96; -5.47) | <0.01 | 88%; 152.70; 40.30; <0.01 |
| Diastolic blood pressure | 17 | 620 | 559 | -6.29 (-8.27; -4.30) | <0.01 | 90%; 160.33; 12.45; <0.01 |
| Heart rate | 10 | 351 | 312 | -5.23 (-9.09; -1.38) | <0.01 | 84%; 57.02; 28.62; <0.01 |
| **Intervention** |  |  |  |  |  |  |
| Including physical postures | | | | | | |
| Systolic blood pressure | 21 | 807 | 822 | -7.19 (-9.61, -4.76) | <0.01 | 88%; 164.91; 22.81; <0.01 |
| Diastolic blood pressure | 20 | 787 | 798 | -5.29 (-6.90, -3.69) | <0.01 | 93%; 271.80; 9.43; <0.01 |
| Heart rate | 13 | 497 | 503 | -4.77 (-7.78, -1.77) | <0.01 | 95%; 259.34; 23.16; <0.01 |
| Not including physical postures | | | | | | |
| Systolic blood pressure | 8 | 233 | 204 | -7.03 (-12.43, -1.63) | 0.01 | 91%; 80.57; 50.02; <0.01 |
| Diastolic blood pressure | 5 | 158 | 128 | -3.94 (-6.09, -1.79) | <0.01 | 75%; 16.20; 3.39; <0.01 |
| Heart rate | 4 | 114 | 107 | -2.31 (-6.27, -1.66) | 0.25 | 40%; 4.96; 6.37; 0.17 |
| **Co-medication** |  |  |  |  |  |  |
| Allowed | | | | | | |
| Systolic blood pressure | 16 | 620 | 537 | -6.50 (-9.90, -3.09) | <0.01 | 89%; 141.93; 38.17; <0.01 |
| Diastolic blood pressure | 13 | 525 | 461 | -4.26 (-6.11, -2.41) | <0.01 | 88%; 96.43; 7.81; <0.01 |
| Heart rate | 8 | 303 | 262 | -1.62 (-3.55; 0.32) | 0.10 | 23%; 9.12; 1.74; 0.24 |
| Not allowed | | | | | | |
| Systolic blood pressure | 3 | 104 | 107 | -10.50 (-23.36, 2.36) | 0.11 | 94%; 31.01; 118.98; <0.01 |
| Diastolic blood pressure | 3 | 104 | 107 | -5.97 (-10.39, -1.54) | <0.01 | 95%; 43.43; 13.21; <0.01 |
| Heart rate | 3 | 104 | 107 | -3.92 (-10.70; -2.87) | 0.26 | 95%; 39.75; 33.71; <0.01 |
| **BP measurement** |  |  |  |  |  |  |
| 24h ABPM | | | | | | |
| Systolic blood pressure | 3 | 118 | 80 | -2.24 (-5.12, 0.65) | 0.13 | 0%; 0.88; 0.00; 0.64 |
| Diastolic blood pressure | 2 | 76 | 56 | -1.94 (-2.77, -1.11) | <0.01 | 0%; 0.59; 0.00; 0.44 |
| Heart rate | 2 | 76 | 56 | 1.17 (0.03; 2.31) | 0.04 | 8%; 1.09; 0.18; 0.30 |
| Clinical | | | | | | |
| Systolic blood pressure | 23 | 922 | 887 | -8.68 (-11.16, -6.19) | <0.01 | 91%; 245.25; 28.02; <0.01 |
| Diastolic blood pressure | 21 | 869 | 835 | -5.41 (-6.93, -3.89) | <0.01 | 93%; 269.21; 9.05; <0.01 |
| Heart rate | 12 | 499 | 487 | -5.67 (-8.24; -3,09) | <0.01 | 85%; 72.91; 12.96; <0.01 |
| **Study origin** |  |  |  |  |  |  |
| Studies from India |  |  |  |  |  |  |
| Systolic blood pressure | 15 | 659 | 647 | -9.30 (-12.21, -6.39) | <0.01 | 93%; 195.52; 26.61; <0.01 |
| Diastolic blood pressure | 14 | 629 | 617 | -5.42 (-7.13, -3.71) | <0.01 | 92%; 172.46; 7.88; <0.01 |
| Heart rate | 8 | 422 | 433 | -5.73 (-8.80; -2.66) | <0.01 | 89%; 62.51; 13.66; <0.01 |
| Studies not from India |  |  |  |  |  |  |
| Systolic blood pressure | 11 | 381 | 320 | -5.85 (-10.02, -1.68) | <0.01 | 82%; 56.40; 37.08; <0.01 |
| Diastolic blood pressure | 9 | 316 | 274 | -4.24 (-6.91, -1.56) | <0.01 | 91%; 93.39; 12.17; <0.01 |
| Heart rate | 6 | 153 | 110 | -2.15 (-7.02; 2.72) | 0.39 | 88%; 40.78; 27.73; <0.01 |

*24h ABPM=24h-ambulatory blood pressure measurement; BP=blood pressure
