# Supplementary material for: A systematic review and meta-analysis of yoga for arterial hypertension
Source: PLoS One. 2025 May 14;20(5):e0323268. doi: 10.1371/journal.pone.0323268 (PMC12077774; doi:10.1371/journal.pone.0323268)
Supplement: S3 Table — (DOCX) [file pone.0323268.s003.docx]

**S3 Table: Subgroup analyses of yoga vs. active control.**

| **Subgroup or outcome** | **No. of studies** | **No. of patients (yoga)** | **No. of patients (usual care)** | **Mean difference (95% confidence interval)** | **P (overall effect)** | **Heterogeneity**  **I^2^; Chi^2^; Tau^2^; P** |
| --- | --- | --- | --- | --- | --- | --- |
| **Participant group** |  |  |  |  |  |  |
| Prehypertension and Hypertension (mixed) | | | | | | |
| Systolic blood pressure | 3 | 93 | 96 | 0.16 (-3.02, 3.34) | 0.92 | 0%; 1.50; 0.00; 0.47 |
| Diastolic blood pressure | 3 | 93 | 96 | -1.34 (-3.81; 1.14) | 0.29 | 0%; 0.70; 0.00; 0.71 |
| Hypertension | | | | | | |
| Systolic blood pressure | 2 | 58 | 59 | -8.22 (-17.97, 1.52) | 0.10 | 89%; 8.81; 43.97; <0.01 |
| Diastolic blood pressure | 2 | 58 | 59 | -2.22 (-4.16; -0.28) | 0.02 | 0%; 0.96; 0.00; 0.33 |
|  |  |  |  |  |  |  |
| **Co-medication** |  |  |  |  |  |  |
| Not allowed | | | | | | |
| Systolic blood pressure | 3 | 85 | 93 | -6.75 (-14.37, 0.86) | 0.08 | 83%; 11.52; 36.60; <0.01 |
| Diastolic blood pressure | 3 | 85 | 93 | -2.20 (-4.07, -0.34) | 0.02 | 0%; 0.97; 0.00; 0.62 |
| **BP measurement** |  |  |  |  |  |  |
| 24h ABPM | | | | | | |
| Systolic blood pressure | 2 | 63 | 66 | -2.79 (-8.48, 2.90) | 0.34 | 0%; 0.00; 0.00; 0.97 |
| Diastolic blood pressure | 2 | 63 | 66 | -2.59 (-6.50, 1.33) | 0.20 | 0%; 0.04; 0.00; 0.84 |
| Clinical | | | | | | |
| Systolic blood pressure | 3 | 88 | 89 | -4.88 (-14.42, 4.66) | 0.32 | 94%; 30.98; 66.07; <0.01 |
| Diastolic blood pressure | 3 | 88 | 89 | -1.76 (-3.42, -0.10) | 0.04 | 0%; 1.78; 0.00; 0.41 |
| **Type of active control** |  |  |  |  |  |  |
| Exercise | | | | | | |
| Systolic blood pressure | 4 | 124 | 121 | -4.41 (-12.18, 3.37) | 0.27 | 90%; 31.51; 55.43; <0.01 |
| Diastolic blood pressure | 4 | 124 | 121 | -1.88 (-3.44, -0.31) | 0.02 | 0%; 1.96; 0.00; 0.58 |
| Heart rate | 2 | 66 | 62 | -5.16 (-8.39, -1.92) | <0.01 | 0%; 0.01; 0.00; <0.01 |

*24h ABPM=24h-ambulatory blood pressure measurement; BP=blood pressure
