# Supplementary material for: A systematic review and meta-analysis of yoga for arterial hypertension
Source: PLoS One. 2025 May 14;20(5):e0323268. doi: 10.1371/journal.pone.0323268 (PMC12077774; doi:10.1371/journal.pone.0323268)
Supplement: S4 Table — (DOCX) [file pone.0323268.s004.docx]

**S4 Table: Subgroup analyses of yoga vs. any control.**

| **Subgroup or outcome** | **No. of studies** | **No. of patients (yoga)** | **No. of patients (usual care)** | **Mean difference (95% confidence interval)** | **P (overall effect)** | **Heterogeneity**  **I^2^; Chi^2^; Tau^2^; P** |
| --- | --- | --- | --- | --- | --- | --- |
| **BP measurement** |  |  |  |  |  |  |
| *24h ABPM* | | | | | | |
| Systolic blood pressure | 5 | 181 | 146 | -2.35 (-4.92, 0.22) | 0.07 | 0%; 0.91; 0.00; 0.92 |
| Diastolic blood pressure | 4 | 139 | 122 | -1.96 (-2.78, -1.15) | <0.01 | 0%; 0.73; 0.00; 0.87 |
| Heart rate | 3 | 112 | 88 | -0.17 (-4.00; 3.67) | 0.93 | 80%; 10.01; 8.96; <0.01 |
| *Clinical* | | | | | | |
| Systolic blood pressure | 25 | 980 | 976 | -8.63 (-11.00, -6.25) | <0.01 | 91%; 266.03; 28.08; <0.01 |
| Diastolic blood pressure | 23 | 927 | 924 | -5.08 (-6.49, -3.67) | <0.01 | 92%; 273.78; 8.58; <0.01 |
| Heart rate | 13 | 529 | 517 | -5.60 (-8.03; -3.18) | <0.01 | 84%; 73.08; 12.25; <0.01 |

*24h ABPM=24h-ambulatory blood pressure measurement; BP=blood pressure
