# Supplementary material for: A systematic review and meta-analysis of yoga for arterial hypertension
Source: PLoS One. 2025 May 14;20(5):e0323268. doi: 10.1371/journal.pone.0323268 (PMC12077774; doi:10.1371/journal.pone.0323268)
Supplement: S5 Table — (DOCX) [file pone.0323268.s005.docx]

**S5 Table: Sensitivity analyses of yoga vs waitlist control.**

| **Subgroup or outcome** | **No. of studies** | **No. of patients (yoga)** | **No. of patients (waitlist)** | **Mean difference (95% confidence interval)** | **P (overall effect)** | **Heterogeneity**  **I^2^; Chi^2^; Tau^2^; P** |
| --- | --- | --- | --- | --- | --- | --- |
| **Diabetic participants excluded** |  |  |  |  |  |  |
| Systolic blood pressure | 23 | 950 | 877 | -7.57 (-9.95, -5.19) | <0.01 | 91%; 232.91; 25.89; <0.01 |
| Diastolic blood pressure | 21 | 878 | 823 | -4.80 (-6.18, -3.42) | <0.01 | 92%; 265.60; 7.29; <0.01 |
| Heart rate | 14 | 575 | 543 | -4.43 (-7.36, -1.50) | <0.01 | 95%; 269.85; 23.76; <0.01 |
| **McCaffrey excluded** |  |  |  |  |  |  |
| Systolic blood pressure | 25 | 1013 | 940 | -7.37 (-9.61, -5.13) | <0.01 | 90%; 230.91; 24.43; <0.01 |
| Diastolic blood pressure | 22 | 918 | 864 | -4.42 (-5.69, -3.16) | <0.01 | 91%; 234.94; 6.24; <0.01 |
| Heart rate | 13 | 548 | 516 | -3.81 (-6.84; -0.79) | 0.01 | 95%; 254.50; 23.19; <0.01 |
| **Diabetic patients and McCaffrey excluded** |  |  |  |  |  |  |
| Systolic blood pressure | 22 | 923 | 850 | -6.91 (-9.23, -4.58) | <0.01 | 90%; 210.08; 23.42; <0.01 |
| Diastolic blood pressure | 20 | 851 | 796 | -4.25 (-5.57, -2.93) | <0.01 | 92%; 227.04; 6.21; <0.01 |
| Heart rate | 13 | 548 | 516 | -3.81 (-6.84; -0.79) | 0.01 | 95%; 254.50; 23.19; <0.01 |
| **Duration of yoga intervention <8 weeks excluded** |  |  |  |  |  |  |
| Systolic blood pressure | 19 | 788 | 717 | -7.37 (-9.93, -4.80) | <0.01 | 89%; 164.04; 23.06; <0.01 |
| Diastolic blood pressure | 18 | 746 | 693 | -4.39 (-5.83, -2.94) | <0.01 | 90%; 178.42; 6.12; <0.01 |
| Heart rate | 11 | 508 | 476 | -4.44 (-7.76; -1.12) | <0.01 | 96%; 268.22; 24.78; <0.01 |
| **Diabetic participants, McCaffrey, yoga intervention <8 weeks excluded** |  |  |  |  |  |  |
| Systolic blood pressure | 17 | 744 | 672 | -5.76 (-8.17, -3.34) | <0.01 | 87%; 124.06; 17.50; <0.01 |
| Diastolic blood pressure | 16 | 702 | 648 | -3.43 (-4.78, -2.09) | <0.01 | 89%; 131.75; 4.50; <0.01 |
| Heart rate | 11 | 508 | 476 | -4.44 (-7.76; -1.12) | <0.01 | 96%; 268.22; 24.78; <0.01 |
