# Supplementary material for: A systematic review and meta-analysis of yoga for arterial hypertension
Source: PLoS One. 2025 May 14;20(5):e0323268. doi: 10.1371/journal.pone.0323268 (PMC12077774; doi:10.1371/journal.pone.0323268)
Supplement: S6 Table — (DOCX) [file pone.0323268.s006.docx]

**S6 Table: Risk of Bias Evaluation**

| **Paper** | **Random sequence generation (selection bias)** | **Reasons for judgement (quote if appropriate)** | **Allocation concealment**  **(selection bias)** | **Reasons for judgement (quote if appropriate)** | **Blinding of participant s and**  **personnel**  **(performan ce bias)** | **Reasons for judgement (quote if appropriate)** | **Blinding of**  **outcome**  **assessm ent**  **(detectio n bias)** | **Reasons for judgement (quote if appropriate)** | **Incomplete outcome data**  **(attrition bias)** | **Reasons for judgement (quote if appropriate)** | **Selective reporting**  **(reporting bias)** | **Reasons for judgement (quote if appropriate)** | **Other bias**  **(result)** | **Other bias**  **-**  **Exclusion**  **Criteria given** | **Reasons for judgement (quote if appropriate)** | **Other bias - Funding** | **Reasons for judgement (quote if appropriate)** |
| --- | --- | --- | --- | --- | --- | --- | --- | --- | --- | --- | --- | --- | --- | --- | --- | --- | --- |
| **Anjana 2022**  Effect of om chanting and yoga nidra on blood pressure and lipid  profile in hypertension e A randomized controlled trial | Low Risk  High Risk  (WK)  Consensus  : Low Risk | Consensus: 80 patients who provided informed written consents were randomly allocated to either intervention group (40) or control group (40) through block randomization method. through block randomization method. (WK)  Consensus was reached between CG and WK by discussing the issue. | Low Risk | Allocation concealment was done in a sequentially numbered opaque and sealed envelope (SNOSE method), handled by a researcher who was not directly involved in the study. | Unclear risk | No information given. | Unclear risk | No information given. | Low Risk | 18.75%  Six patients from the experimental group and nine patients from the control group dropped out of the study. | Low Risk | All outcome data reported. | Low Risk | Low Risk | Exclusion criteria were patients with cardiorespiratory diseases, Neuroendocrine diseases, and patients who are practicing yoga or any form of exercise more than 3 days a week. | Low Risk | No external funding sources were involved in any stage of this study. |
| **Ankolekar et al., 2019**  Role of yoga intervention on quality of life and prehypertension | Unclear Risk | No information given about which method was used for randomisation.    "Subjects with pre-hypertension were divided into control and intervention group by using simple random sampling method." | Unclear Risk | No information given | Unclear Risk | No information given | Unclear Risk | No information given | Unclear Risk | No information given | High Risk | Outcome data missing. No data about prevalence reported.  "Objectives: To assess the prevalence of prehypertension and hypertension among security people." | Unclear Risk | Unclear Risk | No information given | Unclear Risk | No information given |
| **Cohen, D. L. et al., 2011**  Iyengar Yoga versus Enhanced Usual Care on Blood  Pressure  in Patients with Prehypertension to Stage I Hypertension: a Randomized Controlled Trial | Unclear Risk | No information given about which method was used for randomisation.  "Over 23 months, seven cohorts containing 8–12  participants per cohort were recruited sequentially and randomized to either EUC or IY. Because the dropout rate was imbalanced (zero in the EUC arm at this time), randomization was changed from 1 : 1 to 4 : 1 (IY to EUC) for the last cohort to try and balance group numbers." | High Risk | "Although randomized, the trial was unblinded and though we included the EUC control group, it was an active control. While blinding is ideal to eliminate measurement bias, it is not feasible with behavioral interventions." | High Risk | "Although randomized, the trial was unblinded and though we included the EUC control group, it was an active control. While blinding is ideal to eliminate measurement bias, it is not feasible with behavioral interventions." | High Risk | "Although randomized, the trial was unblinded and though we included the EUC control group, it was an active control. While blinding is ideal to eliminate measurement bias, it is not feasible with behavioral interventions." | High Risk | Attrition 27%  Imbalance in IY group (50% Drop Out vs. 3% in EUC group) | Low Risk | All outcome data reported. | Low Risk | Low Risk | "pregnancy or postpartum <3 months; current use of any medications or dietary supplements known to affect BP; body mass index (BMI) >40 kg/m2 ; practice of IY in previous 12 months, or active practice of any other MBT more than two times per month; diabetes mellitus; cardiovascular disease; autonomic neuropathy; current tobacco use; renal insufficiency; and >10 alcoholic drinks per week in women and >15 drinks in men" | Low Risk | "National Institutes of Health grants  R21AT002353-02 (to P.O.S. and  R.R.T.) and M01-RR00040  (General Clinical Research Center) and National Center for  Complementary and Alternative  Medicine (NCCAM)." |
| **Cohen, D. L. et al., 2016**  Blood Pressure Effects of Yoga, Alone or in Combination With  Lifestyle Measures: Results of the Lifestyle  Modification and  Blood Pressure Study (LIMBS) | Low Risk | "After completion of the baseline 24-hour ABP visit, participants were randomized to either the YP, BPEP, or COMBO intervention using a simple (nonstratified), blocked randomization. Small blocks of six were used to avoid imbalances and ensure that no group was off by more than three participants per cohort. Using this system, a biostatistician from the Center for Clinical Epidemiology and Biostatistics at Penn generated a list from which patients were randomized in order of enrollment. Study classes began within 1 to 3 weeks of randomization." | High Risk | "LIMBS II is a phase 2 randomized, nonblinded, prospective, controlled trial to assess the safety and efficacy of a 24-week structured yoga program (YP) vs BP education program (BPEP) vs a combined yoga and education intervention (COMBO)." | High Risk | "LIMBS II is a phase 2 randomized, nonblinded, prospective, controlled trial to assess the safety and efficacy of a 24-week structured yoga program (YP) vs BP education program (BPEP) vs a combined yoga and education intervention (COMBO)." | High Risk | "LIMBS II is a phase 2 randomized, nonblinded, prospective, controlled trial to assess the safety and efficacy of a 24week structured yoga program (YP) vs BP education program (BPEP) vs a combined  yoga and education intervention  (COMBO)." | High Risk | Attrition 34%  "There was a larger than anticipated dropout rate of 34% in the study. A total of 18% of patients dropped out within the first 2 weeks after randomization because of dissatis- faction with group randomization; 66% because of poor attendance, schedule conflicts, or time commit- ments; and 8% because of requiring initiation of drug therapy as a result of SBP >180 mm Hg. (...) Because the dropout rate was high, we felt it was important to do both an intention- to-treat as well as a per-protocol analysis." | Low Risk | All outcome data reported. | Low Risk | Low Risk | ""Pregnancy or postpartum ≤3 months, Currently taking blood pressure–lowering medications or dietary supplements (eg, magnesium, potassium, calcium >1200 mg/d, fish oil, ephedra,  hawthorn, forskolin), Stage 2 hypertension (SBP >160 mm Hg OR diastolic blood pressure  ≥100 mm Hg), Nondominant arm  circumference >50 cm, Body mass index ≥40.0 kg/m2, Practiced yoga >1 time per month in the previous 6 months, Diabetes mellitus or established cardiovascular disease, Known arrhythmias such as atrial flutter or fibrillation or cardiac pacemaker, Any tobacco use (within the previous 30 days), History of chronic kidney disease based on estimated glomerular filtration rate <60 mL/min, Consumption of >7 alcoholic drinks per week (for women) and >14 drinks per week (for men), Known autonomic neuropathy (eg, Shy-Drager syndrome, orthostatic hypotension), Known secondary causes of hypertension (renal artery stenosis, pheochromocytoma, coarctation of aorta, hyperaldosteronism) Use of benzodiazepines, antipsychotic drugs (3-month stable use of selective serotonin reuptake inhibitors allowed), or steroids Known severe musculoskeletal problems such as spinal stenosis that may limit participation in yoga, Practice of other mindbody therapies such as qigong, tai chi, or meditation"" | Low Risk | "The study was funded by the National Center for  Complementary and Alternative  Medicine: 1RO1AT004921-01A1.  The authors have no other conflicts of interest to disclose." |
| **Cramer, H. et al., 2018**  Yoga in Arterial Hypertension | Low Risk | "The patients were stratified by sex and divided by block randomization with randomly varying block sizes into the three following groups (ratio 1 : 1 : 1):  ● An intervention with yoga postures ● An intervention without yoga postures ● A wait list control group.  A biometrician who was not involved in patient recruitment or data acquisition used Random Allocation Software (14) to generate a passwordprotected randomization list to which only he had access. On the basis of this randomization list, opaque envelopes containing the study group allocation were numbered consecutively and sealed. Once a patient had signed the consent form and baseline data acquisition had been concluded, the study physician opened the en- velope with the lowest remaining serial number and the patient was allocated to a group accordingly. At the end of the study, the bio- metrician checked the group allocations and found that all patients had been allocated correctly and according to the randomization list." | Low Risk | "The patients were stratified by sex and divided by block randomization with randomly varying block sizes into the three following groups (ratio 1 : 1 : 1):  ● An intervention with yoga postures ● An intervention without yoga postures ● A wait list control group. A biometrician who was not involved in patient recruitment or data acquisition used Random Allocation Software (14) to generate a passwordprotected randomization list to which only he had access. On the basis of this randomization list, opaque envelopes containing the study group allocation were numbered consecutively and sealed. Once a patient had signed the consent form and baseline data acquisition had been concluded, the study physician opened the en- velope with the lowest remaining serial number and the patient was allocated to a group accordingly. At the end of the study, the bio- metrician checked the group allocations and found that all patients had been allocated correctly and according to the randomization list." | Unclear Risk | No information given | Low Risk | "The person who acquired the data was not involved in patient recruitment, group allocation, or the interven- tions and was blinded to group allocation for the whole duration of the study." | Low Risk | Attrition 11,7%  Five members of the group with yoga postures (20.0%), three of the group without yoga postures (12.0%), and one patient in the control group (4.0%) did not complete the study program (Figure 1; p = 0.22). Therefore, missing values were multiply imputed for six participants at week 12 and nine patients at week 28. | Low Risk | All outcome data reported. | Low Risk | Low Risk | The reasons for exclusion included the following diseases and circumstances:  ●Secondary hypertension  ● Severe psychiatric comorbidities (major depression, dependency disorders, or psychosis)  ●Coronary heart disease, myocardial infarction, pul- monary embolism, or stroke in  the previous 3 months  ●Heart failure of NYHA stage ≥ I  ●Peripheral arterial occlusive disease of stage  ≥ 1  ● Renal failure of stage >2 with glomerular filtration rate (GFR) <60 mL/min/1.73 m2 ● Participation in any other clinical studies at the time of commencement of our trial or planned participation in such studies in the next  28 weeks  ● Pregnancy or breastfeeding. | Low Risk | *This study was supported by the*  *German Occupational Union for*  *Yoga Teachers (Berufsverband der*  *Yogalehrenden in Deutschland, BDY). The sponsor had no influence on the planning or conduct of the study, on the acquisition, management, analysis, or interpretation of the data, on the writing, revision, or approval of the manuscript, or on the decision to submit the manuscript for publication.* |
| **Dhungana, R. R., et al. 2021**  Effects of a health worker-led 3-month yoga intervention on blood pressure of hypertensive patients: a randomised controlled multicentre trial in the primary care setting | Low Risk | "This was a multicentric, two-arm, randomised, wait-list controlled, nonblinded trial comparing structured yoga practice (alongside health education) against health edu- cation only over three months."  "One hundred and twenty-one participants who were on or without medications were  randomised to intervention (n = 61) and wait-list control (n = 60) groups using stratified block randomisation." | Low Risk | "This was a multicentric, two-arm, randomised, wait-list controlled, nonblinded trial comparing structured yoga practice (alongside health education) against health edu- cation only over three months."  "One hundred and twenty-one participants who were on or without medications were randomised to intervention (n = 61) and wait-list control (n = 60) groups using stratified block randomisation." | High Risk | No Blinding.  "This was a multicentric, two-arm, randomised, wait-list controlled, nonblinded trial comparing structured yoga practice (alongside health education) against health edu- cation only over three months." | High Risk | No Blinding.  "The data were collected by the same researcher at baseline and follow-up. The outcome assessors were aware of intervention group allocation." | Low Risk | Attrition: 2,47%  "Data on the primary outcomes were available for a total of 118 participants (Fig. 1). Three participants, two from intervention group (males) and one (female) from con- trol group were lost to follow-up." | Low Risk | All outcome data reported. | Low Risk | Low Risk | "Persons with diabetes, those with a known case of secondary hypertension and/ or other cardiovascular diseases/conditions, pregnant women, and those who practised yoga for 30 days or more in the previous 6 months were excluded." | Low Risk | "RRD received the stipend as a  Ph.D. student from Victoria University, Australia which is funded by the Australian Government Research Training Program Scholarship. The funding body has no role in the design of the study, the collection, analysis, and interpretation of data and in writing the manuscript." |
| **Guamán et al 2022**  EVALUATION OF THE EFFECT OF YOGA  POSTURES ON BLOOD PRESS | High Risk  Unclear  Risk (WK)  Consensus  : High Risk | Consensus: No info about exact randomization method  They were randomly assigned to the yoga group with yoga postures (n = 25), the yoga group without yoga postures (n = 25), or the control group (n = 25)  No information given (WK)  Consensus was reached between CG and WK by discussing the issue. | Unclear risk | No information given. | Unclear risk | study participants were not blinded to the intervention they received.  No information about study personnel (only outcome assessment) | Low Risk | The person acquiring the data was not involved in patient recruitment, group assignment, or interventions and was blinded to group assignment throughout the study. | Low Risk  High Risk (WK)  Consensus:  Low Risk | Consensus: Attrition (as given in paper): 12% n=9  (exact numbers) Attrition: 16% n=12  Five members of the yoga with yoga postures group (20.0%), three members of the yoga without yoga postures group (12.0%) and one patient of the control group (4.0%) did not complete the study program (WK)  Consensus was reached between CG and WK by discussing the issue. | High Risk  Low Risk (WK)  Consensus: High Risk | Consensus: DBP and HR values not reported or labeled as such. All outcome data reported (WK) | Unclear Risk | Low Risk | 1. secondary hypertension  2. Severe psychiatric comorbidities (major depression, dependency disorders or psychosis)  3. Coronary artery disease, myocardial infarction, pulmonary embolism or stroke within the previous 3 months 4. NYHA stage heart failure = I  5.  Peripheral arterial occlusive disease stage =  1  6.                Stage >2 renal failure with glomerular filtration rate (GFR) <60 ml/min/1.73 m2.  7.                Participation in any other clinical studies at the time of the start of our trial or planned participation in such studies in the next 28 weeks 8. Pregnancy or lactation. | Unclear Risk | No information given. |
|  |  |  |  |  |  |  |  |  |  |  |  | Consensus was reached between CG and WK by discussing the issue. |  |  |  |  |  |
| **Hagins, M. et al., 2014**  A Randomized Controlled Trial Comparing the Effects of Yoga  With an Active Control on Ambulatory Blood Pressure in Individuals With Prehypertension and Stage 1 Hypertension | Low Risk | "Coin tosses performed by the primary investigator (MH) were used for sequence generation for treatment group assignment. Sequential results (eg, participant 1 = yoga) were placed inside 90 opaque sealed enve- lopes numbered in advance (eg, 1–90). Once each participant completed pretest measures (with the excep- tion of the survey regarding expectations of treatment efficacy), he/she took the next numbered concealed envelope from within a box located with the measure- ment laboratory." | Low Risk | "Coin tosses performed by the primary investigator (MH) were used for sequence generation for treatment group assignment. Sequential results (eg, participant 1 = yoga) were placed inside 90 opaque sealed enve- lopes numbered in advance (eg, 1–90). Once each participant completed pretest measures (with the excep- tion of the survey regarding expectations of treatment efficacy), he/she took the next numbered concealed envelope from within a box located with the measure- ment laboratory." | High Risk | "All outcome assessors remained blinded to assignment of intervention throughout the study. By necessity for an active intervention, partici- pants were not blinded to intervention assignment." | Low Risk | "All outcome assessors remained blinded to assignment of intervention throughout the study. By necessity for an active intervention, partici- pants were not blinded to intervention assignment." | Low Risk | Attrition 19% | Low Risk | All outcome data reported. | Low Risk | Low Risk | -current use of insulin or oral hypoglycemic agents;  -previous cardiovascular event (prior myocardial infarction, stroke, or angina pectoris);  -current or previous cancer diagnosis;  -congestive heart failure;  -history of kidney disease;  -signs or symptoms of significant peripheral vascular disease;  -significant comorbidities that preclude successful completion of the study (eg, current fractures, Parkinson’s disease, vertigo); -current/regular yoga practitioner (participated in more than 3 yoga sessions within the past year). | Low | There are no competing financial interests in relation to the current work. |
| **Khadka et al 2023**  EFFECT OF YOGA ON CARDIOVASCULAR  AUTONOMIC  REACTIVITY IN ESSENTIAL HYPERTENSIVE PATIENTS | High Risk  Unclear  Risk (WK)  Consensus  : High Risk | Consensus: No info about exact randomization method  Patients were systematically randomized into control and yoga groups  No information given (WK)  Consensus was reached between CG and WK by discussing the issue. | Unclear risk | No information given. | Unclear risk | No information given. | Unclear risk | No information given. | Low Risk | Attrition: 0% | Low Risk | All outcome data reported. | Unclear Risk | Low Risk | Patients with concomitant diseases (diabetes mellitus, secondary and severe hypertension) were excluded from the study. | Unclear Risk | No information given. |
| **Latha et al., 1991**  Yoga, Pranayama, Thermal Biofeedback techniques in the  management of stress and high blood pressure | High risk | "The total number of the subjects were randomly (odd and even number basis) assigned to experimental and control conditions." | Unclear Risk | No information given. | Unclear Risk | No information given | Low Risk | "Physicians checking the BP were unaware of the training programme."  "The questionnaires were administered by another research worker who was unaware of the therapy programme." | High Risk | Attrition 40,9%  "Initially 22 patients were selected satisfying all the criteria. Over a period of 9 months, 9 patients dropped out due to various reasons" | High Risk | Outcome data missing. No baseline BP data of control group mentioned. | Unclear Risk | Unclear Risk | not given | Unclear Risk | No information given |
| **McCaffrey, R. et al., 2014**  The effects of yoga on hypertensive persons in Thailand | Low Risk | "Participants were randomly assigned to ensure homogeneity between experimental and control groups. Participants were matched using Zeller’ s33 minimized randomization (Version 2.01) to control key variables affecting stress and BP, including age, gender, education level, smoking and alcohol use, and exercise habits." | Unclear Risk  Low Risk (WK)  Consensu  s: Unclear  Risk | Consensus: No information about allocation concealment given.  Participants were randomly assigned to ensure homogeneity between experimental and control groups. Participants were matched using Zeller’ s33 minimized randomization (Version 2.01) to control key variables affecting stress and BP, including age, gender, education level, smoking and alcohol use, and exercise habits. (WK)  Consensus was reached between CG and WK by discussing the issue. | Unclear Risk | No information given | Unclear Risk | No information given | Low Risk | "The initial sample consisted of 61 participants; however, at study completion, 7 participants (11%) were dropped. Five participants (experimental group) stopped practicing yoga before program completion due to lack of time and inability to get to the yoga centers. Two control group participants were dropped because one began taking prescribed antihypertensive drugs and another moved out of the province." | Low Risk | All outcome data reported. | Unclear Risk | Unclear Risk | not given | Unclear Risk | No information given |
| **Misra, S. et al., 2019**  Take a deep breath: A randomized control trial of  Pranayama breathing on uncontrolled hypertension | Low Risk | "Eligible participants were randomly assigned to in-class instruction (n = 44), DVD/YouTube group (n = 57), or control (n = 32). For allocation concealment the 133 participants who agreed to participate were listed on an Excel spreadsheet and then assigned to an arm of the study (A, B, or C) by a staff member using Excel random generator. A separate random assignment to the A, B, and C group for mode of instruction (class,  DVD/YouTube, control) was also achieved using  Excel random generator." | Low Risk | "For allocation concealment the 133 participants who agreed to participate were listed on an Excel spreadsheet and then assigned to an arm of the study (A, B, or C) by a staff member using Excel  random generator. A separate random assignment to the A, B, and C group for mode of instruction (class, DVD/YouTube, control) was also achieved using Excel random generator." | Unclear Risk | No information given | Unclear Risk | No information given | High Risk | Attrition 37,6% | High Risk | DBP Scores missing. | Low Risk | Low Risk | - <18 years of age  -chronic obstructive pulmonary disease, chronic renal disease stage II or above, a history of alcoholism, cognitive problems, mental health diagnosis such as schizophrenia, advanced stage congestive heart failure  -non-English speakers  -no access to internet or a DVD player | Low Risk | This study was partially supported from the Agency for Healthcare Research and Quality. The content is solely the responsibility of the authors and does not necessarily represent the official views of the Agency for Healthcare Research and Quality. Clinician’s time for this study was supported by internal funds, Family and Community Medicine Department’s Leader  Explore and Achieve Proficiency (LEAP) programinternal funds. LEAP provides busy clinicians protected time to engage in research activities. |
| **Mourya, M. et al., 2009**  Effect of slow- and fast-breathing exercises on autonomic functions in patients with essential hypertension | Low Risk | "Patients were randomly and equally divided into the control and other two intervention groups, who were advised to do 3 months of slowbreathing and fast-breathing exercises, respectively."  "The yoga instructor and his team asked each subject to pick up one of the 60 slips (20 for each group) to allocate the group." | Low Risk | "The yoga instructor and his team asked each subject to pick up one of the 60 slips (20 for each group) to allocate the group." | High Risk | "The resident and technician involved in recording BP and autonomic functions were unaware of the type of breathing exercise practiced by the patient. The patients were advised not to discuss this with them or with other patients in the laboratory." | Unclear Risk | No information given | Low Risk | Attrition 0% (11,67% excluded from analysis)  "Two (2) patients did not practice slowbreathing exercises regularly and 3 could not learn the technique of fast breathing in spite of all efforts, and 2 patients started dieting. They were not discouraged from visiting the hypertension clinic or breath- ing exercise sessions, but their results are not included in the groups mentioned here." | Low Risk | All outcome data reported. | Low Risk | Low Risk | -age >60 or <20 years  -not stage I hypertension  -normal autonomic function tests  -history of smoking, alcohol or drug intake, receiving drugs that alter the heart rate -already performing breathing or yogic exercises  -patients with secondary hypertension, diabetes mellitus, chronic breathing disorders, congestive heart failure, ischemic heart disease, chronic atrial fibrillation, previous stroke, psychiatric disorder, or clinical evidence of malnutrition | Low Risk | This study was part of an M.D. thesis with no financial support from any agency. There is no financial, personal, political or academic con- flict of interest. |
| **Murugesan, R. et al., 2000**  Effect of selected yogic practices on the management of hypertension | Unclear Risk | No information given about which method was used for randomisation.  "The subjects were divided into three equal groups randomly. " | Unclear Risk | No information given. | Unclear Risk | No information given. | Unclear Risk | No information given | Unclear Risk  Low Risk (WK)  Consensus:  Unclear Risk | Consensus: No information given.  No information given on dropouts? Maybe all completed the study? (WK)  Consensus was reached between CG and WK by discussing the issue. | Low Risk  High Risk  (WK)  Consensu s: Low Risk | Consensus: All outcome data reported.  Outcome measures are probably  complete, but  there is no further information about participants characteristics, group differences, dropouts…  Consensus was reached between CG and WK by discussing the issue. | Unclear Risk | Unclear Risk | No information given | Unclear Risk | No information given |
| **Pandey et al 2023**  Impact of Yoga on Global Cardiovascular Risk as an Add-On to a regular Excercise Regimen in patients with hypertension | High risk  Low Risk (WK)  Consensus  : High Risk | Consensus: No info about exact randomization method  Participants were randomised 1 to 1 into a control group doing standard exercises with a stretching routine and a yoga group who did a yoga routine  Participants were randomised 1 to 1 (WK)  Consensus was reached between CG and WK by discussing the issue. | Unclear risk | No information given. | Unclear risk | No information given. | Unclear risk | No information given. | Unclear Risk  Low Risk (WK)  Consensus:  Unclear Risk | Consensus: No information given.  All participancts completed (WK)  Consensus was reached between CG and WK by discussing the issue. | Low Risk | All outcome data reported. | Low Risk | Low Risk | Patients with symptoms or signs of coronary artery disease and heart failure were excluded. All individuals with known or reported hypertensive end-organ damage, as well as those taking medications or supplements that  affect blood pressure, cholesterol, or inflammation were also excluded. | Low Risk | The authors have no funding sources to declare. |
| **Patil, S. G. et al., 2014**  Effect of yoga on oxidative stress in elderly with grade-i hypertension:  A randomized controlled study | Low Risk | "Selected subjects were randomly divided into yoga group (n=30) and control group (n=30) by using random number table." | Unclear Risk  Low Risk (WK)  Consensu  s: Unclear  Risk | Consensus: No information about allocation concealment given.  Consensus was reached between CG and WK by discussing the issue. | Unclear Risk | No information given. | Low Risk | "Person’s handling data analysis were kept blinded." | Low Risk | Attrition: 5%  (from Flow Chart): absent during postinvestigation: n=3 | Low Risk | All outcome data reported. | Low Risk | Low Risk | Subjects on any medications and subjects with CV risk factors such as diabetes mellitus, hypercholesterolemia and high triglyceride level were excluded from the study. | Low Risk | "We express our sincere thanks to  Department of Science and Technology, Government of India and BLDE University for financial assistance." |
| **Prakash 2015**  To study the role of yoga in management of hypertension | High risk  Low Risk (WK)  Consensus  : High Risk | Consensus: No info about exact randomization method  These patients were randomised into two groups of twenty five each  These patients were randomised into two groups  (WK)  Consensus was reached between CG and WK by discussing the issue. | Unclear risk | No information given. | Unclear risk | No information given. | Unclear risk | No information given. | Unclear risk | No information given. | Low Risk | All outcome data reported. | Unclear Risk | Low Risk | Exclusion criteria for both group included complicated hypertension and those with another associated disease like myocardial disease, stroke, spinal disease, respiratory disease or pregnancy. | Unclear Risk | No information given. |
| **Pushpanatan, P. et al., 2016**  Randomized controlled trial of 12-week yoga therapy as lifestyle  intervention in patients of essential hypertension and cardiac autonomic function tests | Low Risk | Block randomization was used to generate allocation sequence, and serially numbered opaque sealed envelope technique (SNOSE) was applied to allot the subjects either to the control group or to the yoga group after obtaining their written informed consent. | Low Risk | Block randomization was used to generate allocation sequence, and serially numbered opaque sealed envelope technique (SNOSE) was applied to allot the subjects either to the control group or to the yoga group after obtaining their written informed consent. | Unclear Risk | No information given. | Unclear Risk | No information given. | High Risk | Consensus: Attrition: 30% (24 von 80, not balanced between groups)  1 patient drop out in Yoga intervention group  (n=25), n=30 analyzed control group  Consensus was reached by discussing the issue with third scientist (HC) | Low Risk | All outcome data reported. | Unclear Risk | Low Risk | "Subjects with secondary HT, diabetes, ischemic heart disease, nephropathy, retinopathy, and any other chronic illness were also excluded by medical history. Subjects with any physical conditions hindering the performance of yoga practices were excluded." | Unclear Risk | No information given |
|  |  |  |  |  |  |  |  |  | Low Risk (WK) |  |  |  |  |  |  |  |  |
|  |  |  |  |  |  |  |  |  | Consensus: |  |  |  |  |  |  |  |  |
|  |  |  |  |  |  |  |  |  | High Risk |  |  |  |  |  |  |  |  |
| **Saptharishi, L. G. et al., 2009**  Community-based randomized controlled trial of nonpharmacological  interventions in prevention and control of hypertension among young adults | Low Risk | "The remaining 120 subjects were divided into one control and three interventional groups of 30 each, using a standardized randomization process, with a random number generator (SPSS 13.0)." | Unclear Risk  Low Risk (WK)  Consensu  s: Unclear  Risk | Consensus: No information about allocation concealment given.  The remaining 120 subjects were divided into one control and three interventional groups of 30 each, using a standardized randomization process, with a random number generator (SPSS 13.0).  Consensus was reached between CG and WK by discussing the issue. | Unclear Risk | No information given. | Unclear Risk | No information given. | Low Risk  High Risk (WK)  Consensus:  Low Risk | Consensus: Attrition: 9,7%  "Out of the initial 113, 11 subjects (including 6 hypertensives) dropped out.At the end of the study 29, 27, 25 and 21 in the groups I, II, III and IV respectively (102 comprising 33 hypertensives: eight, 12, six and seven in the groups I, II, III and IV respectively) were successfully followed through." | Low Risk | All outcome data reported. | Unclear Risk | Unclear Risk | No information given | Low Risk | "This study was sponsored by the  Indian Council of Medical research  (ICMR), New Delhi under the Short  Term Studentship scheme (STS2007)." |
|  |  |  |  |  |  |  |  |  |  | Control: 29/30; Physical exercise 27/28; |  |  |  |  |  |  |  |
|  |  |  |  |  |  |  |  |  |  | Salt intake 25/28; Yoga 21/27 |  |  |  |  |  |  |  |
|  |  |  |  |  |  |  |  |  |  | Consensus was reached between CG and WK by discussing the issue. |  |  |  |  |  |  |  |
| **Shantakumari et al, 2012** | High Risk | Hypertensive type 2 diabetics in the age group of 35 - 55 years reporting to the Diabetic clinic meeting the inclusion criteria were randomized into two groups based on their number order of visit to the clinic (Figure1). Even numbers formed the control group while odd numbers comprised the experimental group. | Unclear risk | were randomized into two groups based on their number order of visit to the clinic | Unclear Risk | No information given. | Unclear Risk | No information given. | Low Risk | Attrition: 0% | Low Risk | All outcome data reported. | Unclear Risk | Low Risk | Patients who are pregnant, on long term steroids and those with known retinopathy, nephropathy, and coronary artery disease and cerebrovascular diseases were excluded from the study. | Unclear Risk | No information given |
| Effect of a yoga intervention on hypertensive diabetic patients |  |  |  |  |  |  |  |  |  |  |  |  |  |  |  |  |  |
| **Shetty, P. et al., 2017**  Effects of Sheetali and Sheetkari Pranayamas on  Blood Pressure  and Autonomic Function in Hypertensive Patients | Low Risk | "Enrolled participants were randomly allocated either to the intervention group or the wait-list control group. Randomization was accomplished by assigning each participant to a nonidentifying study identification (ID), randomly ordering the IDs, and then using a serial number generator to assign odd or even to each study ID. " | Low Risk | "Enrolled participants were randomly allocated either to the intervention group or the wait-list control group. Randomization was accomplished by assigning each participant to a nonidentifying study identification (ID), randomly ordering the IDs, and then using a serial number generator to assign odd or even to each study ID. " | High Risk | "Group assignments were not concealed from the participants because the intervention was not possible to blind; however, group assignments were not known to the study’s coordinators or technicians collecting or analyzing the data." | Low Risk | "Group assignments were not concealed from the participants because the intervention was not possible to blind; however, group assignments were not known to the study’s coordinators or technicians collecting or analyzing the data." | Low Risk | Attrition: 0%  "No participants were lost to follow-up in this trial, and all participants completed at least 80% of all the study’s visits, based on attendance records from each practice session." | High Risk | DBP Scores missing. | Unclear Risk | Low Risk | Candidates were excluded from participation if they  had: (1) secondary HTN; (2) a history,  symptoms of, and/or  laboratory reports suggestive of renal, neurologic, or  ophthalmologic complications; (3) a history of known  cardiac disease, including coronary artery disease, or  participation in cardiac rehabilitation following bypass  surgery; (4) prior exposure to pranayama practice;  (5) a history of smoking or alcoholism; and (6) difficulty in rolling the tongue for Sheetali practice. | Unclear Risk | No information given. |
| **Shetty et al 2023**  The Role of Integrated Approach to Yoga Therapy-  Based Yoga Module in Improving Cardiovascular  Functions and Lipid Profile in Hypertensive Patients: A Randomized Controlled Trial | Low Risk  High Risk  (WK)  Consensus  : Low Risk | Consensus: Baseline assessments were conducted, after which the participants were randomly allocated to either IG or the control group in a 1:1 ratio. Block randomization technique with varying block lengths was used for this purpose. Randomization was done by a lab technician who was not involved in patient recruitment or assessment using a computerbased random number generator software[21]  Block randomization (WK)  Consensus was reached between CG and WK by discussing the issue. | Low Risk | and prepared sequentially numbered closed envelopes. The study physician opened the envelopes after each participant’s recruitment to reveal their group assignment. | High Risk | Clinical and laboratory variables were evaluated by a physician who was blind to the participant allocation.  It was also not possible to blind the participants. | Unclear Risk | The study physician opened the envelopes after each participant’s recruitment to reveal their group assignment. | Low Risk | Attrition: 7,69% n=5 dropped out | Low Risk | All outcome data reported. | Low Risk | Low Risk | Secondary hypertension  •Severe psychiatric comorbidities (major depression,  dependency disorders, or psychosis)  • Coronary heart disease, myocardial infarction,pulmonary embolism, or stroke in the previous  3 months  • Heart failure of NYHA stage = I  • Peripheral arterial occlusive disease of stage  1  • Renal failure of stage >2 with glomerular  filtration  rate <60 mL/min/1.73 m2  • Participation in any other clinical studies at the time of  commencement of our trial or planned participation in  such studies in the next 12 weeks  • Practiced yoga for 30days or more in the previous  6 months  • Pregnancy or breastfeeding. | Low Risk | This work was supported by Rajiv  Gandhi University of Health  Sciences (Project Code: 17Y002). The sponsor had no influence on the planning or conduct of the study, on the acquisition, management, analysis, or interpretation of the data, on the writing, revision, or approval of the manuscript. |
| **Singh 2022**  Effectiveness Of Yoga and Lifestyle Modification On  Prehypertensive Subjects-A Randomized Controlled  Trial" | Low Risk  High Risk  (WK)  Consensus  : Low Risk | Consensus: Block random sampling was used for allocation of subjects.  Block random sampling was used for allocation of subjects (WK)  Consensus was reached between CG and WK by discussing the issue. | Unclear risk | No information given. | Unclear risk | No information given. | Unclear risk | No information given. | Low Risk | 0% | Low Risk | All outcome data reported. | Low Risk | Low Risk | Exclusion Criteria were Hypertensive patients, adjustments regarding hypertension treatment within the four weeks before the start of the study, Diabetes mellitus, expected inability to understand instructions about Yoga, Lifestyle modification tips and language difficulties/ interpreter needed, Pregnancy or post-partum ≤ 3 months, Body mass index ≥40.0 kg/m2, practiced any mind-body therapies such as Yoga, Tai-Chi or Meditation >1 time per month in the previous 6 months, known secondary causes of hypertension (renal artery stenosis,  hyper-aldosteronism), known arrhythmias such as atrial flutter or fibrillation or cardiac  pacemaker, History of chronic kidney disease , Autonomic neuropathy, orthostatic hypotension, Known severe musculoskeletal problems like spinal stenosis, Use of any anti- psychotic drugs or steroids, Any tobacco use (within the previous 30days), Consumption of >7 alcoholic drinks per week (for women) and >14 drinks per week(for men). | Low Risk | This study received grant from the Department of science and technology (DST- SATYAM)vide SR/SATYAM/238/2016(G) , Govt. of India. |
| **Sujatha, T., Judie, A., 2014**  Effectiveness of a 12-week yoga program on physiopsychological  parameters in patients with hypertension | Unclear Risk | "Of 272 patients, 238 were randomly allocated into two groups: 118 patients (55 males and 63 females) participated in the yoga program group (YP group), and 120 (55 males and 65 females) in the control group (CG)." | Unclear Risk | No information given. | Unclear Risk | No information given. | Unclear Risk | No information given. | Low Risk | Attrition: 0%  (as Table states) Participants analyzed pre/post-intervention n = 238  Not mentioned in text. | Low Risk | All outcome data reported. | Unclear Risk | Low Risk | Exclusion criteria included patients with diabetes mellitus, asthma, and hypercholesteremia, alcoholics, smokers, antenatal and postnatal mothers, and those with regular yoga practice or practicing similar techniques. | Unclear Risk | No information given. |
|  |  |  |  |  |  |  |  |  |  |  |  | BP, HR, weight, height, STAI, |  |  |  |  |  |
|  |  |  |  |  |  |  |  |  |  |  |  | PSS |  |  |  |  |  |
| **Supriya et al 2017**  Yoga training modulates adipokines in adults with high-normal blood pressure and metabolic syndrome | High Risk  Unclear  Risk (WK)  Consensus  : High Risk | Consensus: No info about exact randomization method  Participants were randomly allocated into groups using a computer program.  Participants were randomly allocated into groups using a computer program. (WK)  Consensus was reached between CG and WK by discussing the issue. | Unclear risk | No information given. | Unclear risk | No information given. | Unclear risk | No information given. | Low Risk | 4.4% | High Risk  Low Risk (WK)  Consensu  s: High Risk | Consensus: No post-intervention BP Data.  All outcome data reported  Consensus was reached between CG and WK by discussing the issue. | Low Risk | Low Risk | Participants having symptomatic heart or lung disease, pulmonary illness, severe rheumatoid arthritis or osteoarthri- tis, dementia or mental disorder, previous stroke, severe car- diovascular illness, major orthopedic problems in the lower back, neuromusculo-skeletal illness, and pelvis or lower ex- tremities were excluded. Participants who were on drug ther- apy treating metabolic abnormalities, regular tobacco users, wheelchair users, immobile, with physical conditions not appropriate for yoga exercise were excluded. Additionally, participants who exercised at moderate-tovigorous inten- sity at least 30 minutes per session regularly (3 or more days a week) were also excluded. | Low Risk | This study was supported by the Hong Kong Research Grants Council Hong Kong Ph.D.  Fellowship Scheme (RTVXPF13-  11753), The Hong Kong  Polytechnic University Research Fund (1-ZE17), and The University of Hong Kong Seed Fund for Basic Research. |
| **Thanalakshmi, J. et al., 2020**  Effect of Sheetali pranayama on cardiac autonomic function among  patients with primary hypertension - A randomized controlled trial | Low Risk | "Patients who met the inclusion and exclusion criteria (n 1⁄4 100) were randomly assigned to either HTN with pranayama (Sheetali) group (n 1⁄4 50) or HTN without pranayama group (n 1⁄4 50) using computerized random allocation software version 1." | Low Risk | "Allocation concealment was carried out using sequentially numbered, opaque sealed envelope (SNOSE) method, maintained by a person who was not directly involved in the project who handed over the sealed opaque envelopes to the participants." | High Risk | "Blinding was not possible among the participants as it was a yoga intervention. However, the investigator was blinded to both the group participants, as an independent researcher delivered the pranayama intervention and assessed the HRV." | Low Risk | "Blinding was not possible among the participants as it was a yoga intervention. However, the investigator was blinded to both the group participants, as an independent researcher delivered the pranayama intervention and assessed the HRV." | Low Risk | Attrition: 18%  "One hundred and 75 patients were screened for the study, out of which 100 patients met the inclusion and exclusion criteria, and were recruited for the study. Ten patients in HTN with pranayama and eight patients in HTN without pranayama group did not complete the study and the reasons for the dropout also depicted in Fig. 1." | Low Risk | All outcome data reported.  BP, HRV | Unclear Risk | Low Risk | Patients with history of major  cardiac/renal/pulmonary disease or history of any surgery in the recent past or history of major medical illness in the past  such as tuberculosis, diabetes mellitus, and  bronchial asthma were excluded from the study. | Unclear Risk | No information given. |
| **Thiyagarajan, R. et al., 2015**  Additional benefit of yoga to standard lifestyle modification on blood pressure in prehypertensive subjects: a randomized controlled study | Low Risk | "The prehypertensive subjects were randomized into LSM group and LSMþ yoga group. Block randomization method was used to generate the allocation sequence with the ratio of 1:1 between LSM and yoga groups. Individuals were allotted to either LSM or yoga group using the serially numbered opaque sealed envelope technique." | High Risk | "The allocation list was prepared by one of the co-authors. The first author of this study enrolled  and assigned subjects to the groups. " | High Risk | "This is a parallel active-controlled, randomized, unblinded study."  "As the comparatoragent (LSM intervention) was active controlled, neither the subjects nor the yoga teachers were blinded." | Unclear Risk | No information given | High Risk  Low Risk (WK)  Consensus:  High Risk | Consensus: Attrition: 47,9%  "A total of 49 prehypertensive subjects completed LSM intervention and 51 completed LSM þ yoga intervention with the dropout rate of 46% and 43%, respectively."  no huge group differences, High drop-out-rate. We included only those subjects who had completed 12 weeks of the  particular intervention for statistical analysis  (per protocol analysis). A  total of 49 prehypertensive subjects completed LSM intervention and 51  completed LSMþyoga intervention with the dropout rate of 46% and 43%, respectively.  Consensus was reached between CG and WK by discussing the issue. | Low Risk | All outcome data reported.  "Addition of yoga to standard LSM was expected to produce additional benefits in terms of reduction in the BP. Statistically significant difference in systolic  BP between LSM and LSMþyoga groups was kept as primary end point of the study." | Low Risk | Low Risk | (history of chronic illness, CVDs, diabetes, primary autonomic insufficiency, kidney diseases, sports person, under medication for prehypertension and chronic disease, no written consent | Low Risk | "The Department of Science and Technology, New Delhi, India, funded the first author through  INSPIRE fellowship." |
| **Tolbaños Roche, L., Mas Hesse, B., 2014**  Application of an integrative yoga therapy programme in cases of  essential arterial hypertension in public healthcare | Unclear Risk | "They were randomly selected from a list which had been created, also randomly, by the team of physicians of the health centre. 25 patients were assigned to each group (study group and control group) by systematic random sampling." | Unclear  Risk (CG)  Low Risk (WK)  Consensu  s: Unclear  Risk | Consensus: No information about allocation concealment given.  They were randomly selected from a  list which had been created, also randomly, by the team of physicians  of the health centre. 25 patients were assigned to each group  (study group and control group) by systematic random sampling.  After appointments with each of these initial participants, they  were randomly selected from the list until the consent of participation  in the study was obtained from 20 patients in each group (WK)  Consensus was reached by discussing the issue with third scientist (HC) | Unclear Risk | No information given. | Unclear Risk | No information given. | High Risk  Low Risk (WK)  Consensus:  High Risk | Consensus: Attrition: 60%  "In the study group 10 participants completed the study and in the control group, even though  14 completed the study, four had to be excluded due to changes in diagnosis and medication, leaving a total of 10 participants."  no huge group differences, High drop-out-rate. 20 patients in each group. In the study group 10 participants completed the study and in the control group, even though 14 completed the study, four had to be excluded due to changes in diagnosis and medication, leaving a total of 10 participants.  Consensus was reached between CG and WK by discussing the issue. | Low Risk | All outcome data reported.  Blood pressure, HADS, PANAS, SISR3 pre/post-intervention reported | High Risk (CG)  Unclear  Risk (WK)  Consensu  s: Unclear  Risk | Unclear Risk | Consensus: info not given  change in BP medication or diagnosis ("...four had to be excluded due to changes in diagnosis and medication...") (CG)  Consensus was reached by discussing the issue with third scientist (HC) | Low Risk | "There is no financial conflict of interest." |
| **Tolbaños Roche, L. 2017**  YOGA and self-regulation in management of essential arterial hypertension and associated emotional symptomatology: A randomized controlled trial | High risk | No information about exact randomization method  "Following a randomly selection from an essential arterial hy- pertension patients list, which has been created, also randomly, by the medical team of the Health Centre, was carried out. Twenty five participants were assigned to each group: HT Meditation, Pra- nayama, Yoga Practice and Control, by systematic random sam- pling." | Unclear Risk | No information given. | Unclear Risk | No information given. | Unclear Risk | No information given. | High Risk | Attrition: 35,3%  "The initial sample of the study consisted of  100 participants (...). Twenty five participants were assigned to each group: HT Meditation, Pra- nayama, Yoga Practice and Control, by systematic random sam- pling. The signed consent to participate in the study was given by 85 participants (HT Meditation: 21; Pranayama:  23; Yoga Practice: 22 and Control: 19). Finally, 55 participants completed the study: HT  Meditation: 12; Pranayama: 19, Yoga Practice:  14 and Control: 10." | Low Risk | All outcome data reported. | Unclear Risk | Unclear Risk | No information given | Low Risk | "Funding None.  Conflict of interest  The authors confirm that there are no known conflicts of interest." |
| **Wahyuni, N. et al., 2020**  The effectiveness of yoga exercise toward blood pressure and endothelial-derived hyperpolarizing factor  (Edhf) level in hypertensive diabetic population | Low Risk | "This research was a randomized control trial that involved 39 hypertensive diabetic respondents and was conducted in Denpasar, Bali, Indonesia."  "This research involved 39 respondents who grouped using a simple random sampling method with a random number table. (...) The study involved two arms (yoga group and control group). Respondents were divided into two groups randomly. Blocked randomization was used to group the respondents. Blocked randomization was used to group the respondents. The block size was not stated in the protocol, so the investigators were blinded." | Low Risk | "This research was a randomized control trial that involved 39 hypertensive diabetic respondents  and was conducted in Denpasar, Bali, Indonesia."  "This research involved 39 respondents who grouped using a simple random sampling method with a random number table. (...) The study involved two arms (yoga group and control group). Respondents were divided into two groups randomly. Blocked randomization was used to group the respondents. Blocked randomization was used to group the  respondents. The block size was not stated in the protocol, so the investigators were blinded." | Unclear Risk | No information given about blinding of participants.  "The block size was not stated in the protocol, so the investigators were blinded." | Unclear Risk | No information | Low Risk | Attrition: 11,76%  "Respondents were dropped out when they were unable to complete the treatment given, suffered injuries during the study, or resigned by themselves." n=4 discontinued | Low Risk | All outcome data reported. | Low Risk | Low Risk | Respondents were excluded if they have the following events:  has emergency hypertension during the exercise, participate in a physical  exercise program other than the intervention, have  a history or are currently experiencing injuries, have physical disabilities, or have balance disorders.  Respondents were dropped out when they were  unable to complete the treatment given, suffered  injuries during the study, or resigned by themselves. | Low Risk | "The authors would like to thank  Udayana University Institute for  Research and Community Service  (LPPM) for research funding." |
| **Wolff, M. et al., 2016**  Impact of a short home-based yoga programme on blood pressure in  patients with hypertension: a randomized controlled trial in primary care | Low Risk | "To ensure allocation concealment, randomization to groups was undertaken by a research assistant not involved in recruitment using a computer-generated random number schedule with block size of four. " | Low risk | "To ensure allocation concealment, randomization to groups was undertaken by a research assistant not involved in recruitment using a computer-generated random number schedule with block size of four. " | Unclear Risk | No information about blinding of participants. | Low Risk | "The physical assessments at baseline and follow-up were conducted by trained nurses and care assistants who remained blinded to group allocation throughout the study." | Low Risk | Attrition: 10,5%  Intervention group: Lost to follow-up (n=11: unable to attend follow-up n=3, withdrew n=4, illness n=1, no stated reason n=3)  Control group: Lost to follow-up (n=9: unable to attend follow-up n=2, illness n=5, no stated reason n=2) | Low Risk | All outcome data reported.  Objectives: "The primary outcome was change in BP. The key secondary outcome was selfrated QOL (World Health  Organization Quality of Life  Assessment, WHOQOL-BREF). 21 Other secondary outcomes included stress (Perceived Stress Scale, PSS),22 depression and anxiety (Hospital Anxiety and Depression Scale, HADS).2" | Low Risk | Low Risk | Exclusion criteria included BP measurements at  baseline control outside the range of 120–180  (systolic) or 80–110 mm Hg  (diastolic); that is, below the definitions for  optimal or above those for  grade 3 hypertension, respectively. Patients requiring ongoing adjustment  of BP medication during the 4 weeks before baseline were also excluded.  Patients with expected inability to understand instructions about the yoga  exercises, physical or mental incapacity to carry out yoga exercises,  or language problems/interpreter needs were also excluded. Aside from  the above there were no medical exclusion criteria. | Low Risk | "This study was funded by the  Faculty of Medicine at Lund University, the Ekhaga Foundation, the Swedish Heart-Lung Foundation and the Swedish  Southern Health Care Region, Agreement for Medical Education and Research (ALF) funding from Region Skåne and a Swedish  Research Council grant awarded to  Kristina Sundquist." |
| **Caption/Explanatory notes:**  CG = Dr. med. univ. Christoph Geiger  WK = Dr. med. Wiebke Kohl-Heckl  HC = Prof. Dr. Holger Cramer    Discarded assessment in red | | | | | | | | | | | | | | | | | |

Linear regression test of funnel plot asymmetry

**Supplementary Figure S1:** Funnel plot of yoga vs usual care for SBP.

Linear regression test of funnel plot asymmetry

**Supplementary Figure S2:** Funnel plot of yoga vs usual care for DBP.

Linear regression test of funnel plot asymmetry

**Supplementary Figure S3:** Funnel plot of yoga vs usual care for HR.

**Supplementary Figure S4:** Bubble plot, univariate meta-regression of yoga vs usual care for SBP.

**Supplementary Figure S5:** Bubble plot, univariate meta-regression of yoga vs usual care for DBP.

**Supplementary Figure S6:** Bubble plot, univariate meta-regression of yoga vs usual care for HR
