# Supplementary material for: A systematic review and meta-analysis of yoga for arterial hypertension
Source: PLoS One. 2025 May 14;20(5):e0323268. doi: 10.1371/journal.pone.0323268 (PMC12077774; doi:10.1371/journal.pone.0323268)
Supplement: S7 Table — (DOCX) [file pone.0323268.s007.docx]

**S7 Table: Quality of Evidence Assessment**

| **Effect of Yoga on SBP (Yoga vs. waitlist)** | **Study type** | **Weight (%)** | **Overall Risk of Bias** | **Inconsistency**  subgroup analyses did not diminish heterogeneity. thus it remains high | **Indirectness**  some variation due to population differences (diabetic patients), some variation due to type of BP measurement | **Imprecision**  for continuous outcomes a population of n < 400 participants is considered imprecise | **Publication bias**  In funnel plots visual asymmetry was present suggesting publication bias. Using Egger’s test, no publication bias could be identified. | **Overall quality of evidence**  For this outcome very low quality of evidence was found. |
| --- | --- | --- | --- | --- | --- | --- | --- | --- |
| **Anjana 2022** Effect of om chanting and yoga nidra on blood pressure and lipid profile in hypertension: A randomized controlled trial | RCT | 4,9 | some concern |  |  |  |  |  |
| **Ankolekar et al., 2019** Role of yoga intervention on quality of life and prehypertension | RCT | 4,8 | high |  |  |  |  |  |
| **Cohen, D. L. et al., 2011** Iyengar Yoga versus Enhanced Usual Care on Blood Pressure in Patients with Prehypertension to Stage I Hypertension: a Randomized Controlled Trial | RCT | 3,4 | high |  | ABPM |  |  |  |
| **Cramer, H. et al., 2018** Yoga in Arterial Hypertension | RCT | 4,4 | some concern |  | ABPM |  |  |  |
| **Dhungana, R. R., et al. 2021** Effects of a health worker-led 3-month yoga intervention on blood pressure of hypertensive patients: a randomised controlled multicentre trial in the primary care setting | RCT | 4,5 | high |  |  |  |  |  |
| **Guamán et al 2022**  Evaluation of the effect of Yoga postures on Blood pressure | RCT | 4,2 | high |  | ABPM |  |  |  |
| **Khadka et al 2023**  Effect of Yoga on cardiovascular autonomic reactivity in essential hypertensive patients | RCT | 4,4 | high |  |  |  |  |  |
| **Latha et al., 1991** Yoga, Pranayama, Thermal Biofeedback techniques in the management of stress and high blood pressure | RCT | 2,3 | high |  |  |  |  |  |
| **McCaffrey, R. et al., 2014** The effects of yoga on hypertensive persons in Thailand | RCT | 3,1 | some concern |  |  |  |  |  |
| **Misra, S. et al., 2019** Take a deep breath: A randomized control trial of Pranayama breathing on uncontrolled hypertension | RCT | 2,3 | high |  | diabetic patients |  |  |  |
| **Mourya, M. et al., 2009** Effect of slow- and fast-breathing exercises on autonomic functions in patients with essential hypertension | RCT | 4,7 | some concern |  |  |  |  |  |
| **Murugesan, R. et al., 2000** Effect of selected yogic practices on the management of hypertension | RCT | 2,9 | some concern |  |  |  |  |  |
| **Prakash 2015** To study the role of yoga in management of hypertension | RCT | 2,4 | high |  |  |  |  |  |
| **Pushpanatan, P. et al., 2016** Randomized controlled trial of 12-week yoga therapy as lifestyle intervention in patients of essential hypertension and cardiac autonomic function tests | RCT | 3,8 | some concern |  |  |  |  |  |
| **Saptharishi, L. G. et al., 2009** Community-based randomized controlled trial of non-pharmacological interventions in prevention and control of hypertension among young adults | RCT | 4,2 | some concern |  |  |  |  |  |
| **Shantakumari et al, 2012** Effect of a yoga intervention on hypertensive diabetic patients | RCT | 4,4 | high |  | diabetic patients |  |  |  |
| **Shetty, P. et al., 2017** Effects of Sheetali and Sheetkari Pranayamas on Blood Pressure and Autonomic Function in Hypertensive Patients | RCT | 4,5 | high |  |  |  |  |  |
| **Shetty et al 2022** The Role of Integrated Approach to Yoga Therapy-Based Yoga Module in Improving Cardiovascular Functions and Lipid Profile in Hypertensive Patients: A Randomized Controlled Trial | RCT | 4,2 | high |  |  |  |  |  |
| **"Singh 2022** Effectiveness Of Yoga and Lifestyle Modification On Prehypertensive Subjects-A Randomized Controlled Trial" | RCT | 5,1 | some concern |  |  |  |  |  |
| **Sujatha, T., Judie, A., 2014** Effectiveness of a 12-week yoga program on physiopsychological parameters in patients with hypertension | RCT | 4,8 | some concern |  |  |  |  |  |
| **Thanalakshmi, J. et al., 2020** Effect of Sheetali pranayama on cardiac autonomic function among patients with primary hypertension - A randomized controlled trial | RCT | 3,8 | high |  |  |  |  |  |
| **Thiyagarajan, R. et al., 2015** Additional benefit of yoga to standard lifestyle modification on blood pressure in prehypertensive subjects: a randomized controlled study | RCT | 4,9 | high |  |  |  |  |  |
| **Tolbaños Roche, L., Mas Hesse, B., 2014** Application of an integrative yoga therapy programme in cases of essential arterial hypertension in public healthcare | RCT | 2,1 | high |  |  |  |  |  |
| **Tolbaños Roche, L. 2017** YOGA and self-regulation in management of essential arterial hypertension and associated emotional symptomatology: A randomized controlled trial | RCT | 2,4 | high |  |  |  |  |  |
| **Wahyuni, N. et al., 2020** The effectiveness of yoga exercise toward blood pressure and endothelial-derived hyperpolarizing factor  (Edhf) level in hypertensive diabetic population | RCT | 3,2 | some concern |  | diabetic patients |  |  |  |
| **Wolff, M. et al., 2016** Impact of a short home-based yoga programme on blood pressure in patients with hypertension: a randomized controlled trial in primary care | RCT | 4,4 | some concern |  |  |  |  |  |
|  |  |  | **very serious -2** | **some -1** | **some -1** | **none 0** | **none 0** | **very low** |
| **Effect of Yoga on SBP (Yoga vs. active control)** | **Study type** | **Weight (%)** | **Overall Risk of Bias** | **Inconsistency**  subgroup analyses did not diminish heterogeneity. thus it remains high | **Indirectness**  some variation due to control intervention differences (exercise vs. health education), type of BP measurement | **Imprecision**  for continuous outcomes a population of n < 400 participants is considered imprecise | **Publication bias**  In funnel plots visual asymmetry was present suggesting publication bias. Using Egger’s test, no publication bias could be identified. | **Overall quality of evidence**  For this outcome very low quality of evidence was found. |
| **Cohen, D. L. et al., 2016** Blood Pressure Effects of Yoga, Alone or in Combination With Lifestyle Measures: Results of the Lifestyle Modification and Blood Pressure Study (LIMBS) | RCT | 17,6 | high |  | ABPM health education |  |  |  |
| **Hagins, M. et al., 2014** A Randomized Controlled Trial Comparing the Effects of Yoga  With an Active Control on Ambulatory Blood Pressure  in Individuals With Prehypertension and Stage 1 Hypertension | RCT | 17,6 | high |  | ABPM exercise |  |  |  |
| **Pandey et al 2023** Impact of Yoga on Global Cardiovascular Risk as an Add-On to a regular Excercise Regimen  in patients with hypertension. | RCT | 20,4 | high |  | exercise |  |  |  |
| **Patil, S. G. et al., 2014** Effect of yoga on oxidative stress in elderly with grade-i hypertension:  A randomized controlled study | RCT | 22,3 | some concern |  | exercise |  |  |  |
| **Saptharishi, L. G. et al., 2009** Community-based randomized controlled trial of non-pharmacological  interventions in prevention and control of hypertension  among young adults | RCT | 22,1 | some concern |  | exercise |  |  |  |
|  |  |  | **very serious -2** | **none 0** | **some -1** | **very serious -2** | **none 0** | **very low** |
| **Effect of Yoga on DBP (Yoga vs. waitlist control)** | **Study type** | **Weight (%)** | **Overall Risk of Bias** | **Inconsistency**  subgroup analyses did not diminish heterogeneity. thus it remains high | **Indirectness**  some variation due to population differences (diabetic patients), some variation due to type of BP measurement | **Imprecision**  for continuous outcomes a population of n < 400 participants is considered imprecise | **Publication bias**  In funnel plots visual asymmetry was present suggesting publication bias. Using Egger’s test, no publication bias could be identified. | **Overall quality of evidence**  For this outcome very low quality of evidence was found. |
| **Anjana 2022** Effect of om chanting and yoga nidra on blood pressure and lipid profile in hypertension e A randomized controlled trial | RCT | 5,9 | some concern |  |  |  |  |  |
| **Ankolekar et al., 2019** Role of yoga intervention on quality of life and prehypertension | RCT | 5,6 | high |  |  |  |  |  |
| **Cohen, D. L. et al., 2011** Iyengar Yoga versus Enhanced Usual Care on Blood Pressure in Patients with Prehypertension to Stage I Hypertension: a Randomized Controlled Trial | RCT | 6,1 | high |  | ABPM |  |  |  |
| **Cramer, H. et al., 2018** Yoga in Arterial Hypertension | RCT | 3,8 | some concern |  | ABPM |  |  |  |
| **Dhungana, R. R., et al. 2021** Effects of a health worker-led 3-month yoga intervention on blood pressure of hypertensive patients: a randomised controlled multicentre  trial in the primary care setting | RCT | 5,2 | high |  |  |  |  |  |
| **Khadka et al 2023**  Effect of Yoga on cardiovascular autonomic reactivity in essential hypertensive patients | RCT | 6 | high |  |  |  |  |  |
| **Latha et al., 1991** Yoga, Pranayama, Thermal Biofeedback techniques in the  management of stress and high blood pressure | RCT | 1,3 | high |  |  |  |  |  |
| **McCaffrey, R. et al., 2014** The effects of yoga on hypertensive persons in Thailand | RCT | 3,1 | some concern |  |  |  |  |  |
| **Mourya, M. et al., 2009** Effect of slow- and fast-breathing exercises on  autonomic functions in patients with essential hypertension | RCT | 6 | high |  |  |  |  |  |
| **Murugesan, R. et al., 2000** Effect of selected yogic practices on the management of hypertension | RCT | 1,9 | some concern |  |  |  |  |  |
| **Prakash 2015** To study the role of yoga in management of hypertension | RCT | 2,8 | high |  |  |  |  |  |
| **Pushpanatan, P. et al., 2016** Randomized controlled trial of 12-week yoga therapy as lifestyle  intervention in patients of essential hypertension and  cardiac autonomic function tests | RCT | 3,8 | some concern |  |  |  |  |  |
| **Saptharishi, L. G. et al., 2009** Community-based randomized controlled trial of non-pharmacological  interventions in prevention and control of hypertension  among young adults | RCT | 4,6 | some concern |  |  |  |  |  |
| **Shantakumari et al, 2012** Effect of a yoga intervention on  hypertensive diabetic patients | RCT | 4,8 | high |  | diabetic patients | |  |  |
| **Shetty et al 2022** The Role of Integrated Approach to Yoga Therapy-Based Yoga Module in Improving Cardiovascular Functions and Lipid Profile in Hypertensive Patients: A Randomized Controlled Trial | RCT | 4,8 | high |  |  |  |  |  |
| **"Singh 2022** Effectiveness Of Yoga and Lifestyle Modification On Prehypertensive Subjects-A Randomized Controlled Trial" | RCT | 6,2 | some concern |  |  |  |  |  |
| **Sujatha, T., Judie, A., 2014** Effectiveness of a 12-week yoga program on physiopsychological  parameters in patients with hypertension | RCT | 5,7 | some concern |  |  |  |  |  |
| **Thanalakshmi, J. et al., 2020** Effect of Sheetali pranayama on cardiac autonomic function among  patients with primary hypertension - A randomized controlled trial | RCT | 4,4 | high |  |  |  |  |  |
| **Thiyagarajan, R. et al., 2015** Additional benefit of yoga to standard lifestyle modification on  blood pressure in prehypertensive subjects:  a randomized controlled study | RCT | 5,9 | high |  |  |  |  |  |
| **Tolbaños Roche, L., Mas Hesse, B., 2014** Application of an integrative yoga therapy programme in cases of  essential arterial hypertension in public healthcare | RCT | 1,5 | high |  |  |  |  |  |
| **Tolbaños Roche, L. 2017** YOGA and self-regulation in management of essential arterial hypertension and associated emotional symptomatology: A randomized controlled trial | RCT | 1,9 | high |  |  |  |  |  |
| **Wahyuni, N. et al., 2020** The effectiveness of yoga exercise toward blood  pressure and endothelial-derived hyperpolarizing factor  (Edhf) level in hypertensive diabetic population | RCT | 3,5 | some concern |  | diabetic patients |  |  |  |
| **Wolff, M. et al., 2016** Impact of a short home-based yoga programme on blood pressure in  patients with hypertension: a randomized controlled trial in primary care | RCT | 5,3 | some concern |  |  |  |  |  |
|  |  |  | **very serious -2** | **some -1** | **some -1** | **none 0** | **none 0** | **very low** |
| **Effect of Yoga on DBP (Yoga vs. active control)** | **Study type** | **Weight (%)** | **Overall Risk of Bias** | **Inconsistency**  subgroup analyses did not diminish heterogeneity. thus it remains high | **Indirectness**  some variation due to control intervention differences (exercise vs. health education), type of BP measurement | **Imprecision**  for continuous outcomes a population of n < 400 participants is considered imprecise | **Publication bias**  In funnel plots visual asymmetry was present suggesting publication bias. Using Egger’s test, no publication bias could be identified. | **Overall quality of evidence**  For this outcome very low quality of evidence was found. |
| **Cohen, D. L. et al., 2016** Blood Pressure Effects of Yoga, Alone or in Combination With Lifestyle Measures: Results of the Lifestyle Modification and Blood Pressure Study (LIMBS) | RCT | 5 | high |  | ABPM health education |  |  |  |
| **Hagins, M. et al., 2014** A Randomized Controlled Trial Comparing the Effects of Yoga  With an Active Control on Ambulatory Blood Pressure  in Individuals With Prehypertension and Stage 1 Hypertension | RCT | 10,3 | high |  | ABPM exercise |  |  |  |
| **Pandey et al 2023** Impact of Yoga on Global Cardiovascular Risk as an Add-On to a regular Excercise Regimen  in patients with hypertension. | RCT | 14,2 | high |  | exercise |  |  |  |
| **Patil, S. G. et al., 2014** Effect of yoga on oxidative stress in elderly with grade-i hypertension:  A randomized controlled study | RCT | 47,8 | some concern |  | exercise |  |  |  |
| **Saptharishi, L. G. et al., 2009** Community-based randomized controlled trial of non-pharmacological  interventions in prevention and control of hypertension  among young adults | RCT | 22,8 | some concern |  | exercise |  |  |  |
|  |  |  | **very serious -2** | **none 0** | **some -1** | **very serious -2** | **none 0** | **very low** |
| **Effect of Yoga on HR (Yoga vs. waitlist control)** | **Study type** | **Weight (%)** | **Overall Risk of Bias** | **Inconsistency**  subgroup analyses did not diminish heterogeneity. thus it remains high | **Indirectness**  some variation due to type of BP measurement | **Imprecision**  for continuous outcomes a population of n < 400 participants is considered imprecise | **Publication bias**  In funnel plots visual asymmetry was present suggesting publication bias. Using Egger’s test, no publication bias could be identified. | **Overall quality of evidence**  For this outcome very low quality of evidence was found. |
| **Cohen, D. L. et al., 2011** Iyengar Yoga versus Enhanced Usual Care on Blood Pressure  in Patients with Prehypertension to Stage I Hypertension:  a Randomized Controlled Trial | RCT | 9,3 | high |  | ABPM |  |  |  |
| **Cramer, H. et al., 2018** Yoga in Arterial Hypertension | RCT | 8 | some concern |  | ABPM |  |  |  |
| **Khadka et al 2023** EFFECT OF YOGA ON CARDIOVASCULAR AUTONOMIC REACTIVITY IN ESSENTIAL HYPERTENSIVE PATIENTS | RCT | 7 | high |  |  |  |  |  |
| **McCaffrey, R. et al., 2014** The effects of yoga on hypertensive persons in Thailand | RCT | 8,1 | some concern |  |  |  |  |  |
| **Murugesan, R. et al., 2000** Effect of selected yogic practices on the management of hypertension | RCT | 5,5 | some concern |  |  |  |  |  |
| **Pushpanatan, P. et al., 2016** Randomized controlled trial of 12-week yoga therapy as lifestyle  intervention in patients of essential hypertension and  cardiac autonomic function tests | RCT | 7,4 | some concern |  |  |  |  |  |
| **Shetty, P. et al., 2017** Effects of Sheetali and Sheetkari Pranayamas on Blood Pressure  and Autonomic Function in Hypertensive Patients | RCT | 6,5 | high |  |  |  |  |  |
| **Shetty et al 2022** The Role of Integrated Approach to Yoga Therapy-Based Yoga Module in Improving Cardiovascular Functions and Lipid Profile in Hypertensive Patients: A Randomized Controlled Trial | RCT | 5,9 | high |  |  |  |  |  |
| **"Singh 2022** Effectiveness Of Yoga and Lifestyle Modification On Prehypertensive Subjects-A Randomized Controlled Trial" | RCT | 9,4 | some concern |  |  |  |  |  |
| **Sujatha, T., Judie, A., 2014** Effectiveness of a 12-week yoga program on physiopsychological  parameters in patients with hypertension | RCT | 9,2 | some concern |  |  |  |  |  |
| **Thanalakshmi, J. et al., 2020** Effect of Sheetali pranayama on cardiac autonomic function among  patients with primary hypertension - A randomized controlled trial | RCT | 6,9 | high |  |  |  |  |  |
| **Thiyagarajan, R. et al., 2015** Additional benefit of yoga to standard lifestyle modification on  blood pressure in prehypertensive subjects:  a randomized controlled study | RCT | 8,4 | high |  |  |  |  |  |
| **Tolbaños Roche, L., Mas Hesse, B., 2014** Application of an integrative yoga therapy programme in cases of  essential arterial hypertension in public healthcare | RCT | 4,4 | high |  |  |  |  |  |
| **Tolbaños Roche, L. 2017** YOGA and self-regulation in management of essential arterial hypertension and associated emotional symptomatology: A randomized controlled trial | RCT | 4,1 | high |  |  |  |  |  |
|  |  |  | **very serious -2** | **some -1** | **some -1** | **none 0** | **none 0** | **very low** |
| **Effect of Yoga on HR (Yoga vs. active control)** | **Study type** | **Weight (%)** | **Overall Risk of Bias** | **Inconsistency**  no subgroup analysis performed | **Indirectness**  some variation due to control intervention differences (exercise vs. health education), type of BP measurement | **Imprecision**  for continuous outcomes a population of n < 400 participants is considered imprecise | **Publication bias**  no evaluation of publication bias performed | **Overall quality of evidence**  For this outcome very low quality of evidence was found. |
| **Hagins, M. et al., 2014** A Randomized Controlled Trial Comparing the Effects of Yoga  With an Active Control on Ambulatory Blood Pressure  in Individuals With Prehypertension and Stage 1 Hypertension | RCT | 63 | high |  | ABPM health education |  |  |  |
| **Pandey et al 2023** Impact of Yoga on Global Cardiovascular Risk as an Add-On to a regular Excercise Regimen  in patients with hypertension. | RCT | 37 | high |  | exercise |  |  |  |
|  |  |  | **very serious -2** | **very serious -2** | **very serious -2** | **very serious -2** | **very serious -2** | **very low** |
|  |  |  |  |  |  |  |  |  |
|  |  |  |  |  |  |  |  |  |
