# Supplementary material for: A systematic review and meta-analysis of yoga for arterial hypertension
Source: PLoS One. 2025 May 14;20(5):e0323268. doi: 10.1371/journal.pone.0323268 (PMC12077774; doi:10.1371/journal.pone.0323268)
Supplement: S8 Table — (DOCX) [file pone.0323268.s008.docx]

**S8 Table: Results of Quality of Evidence Assessment**

| **Concern** | **Downgrade for rating of evidence (Upgrade not common)** |
| --- | --- |
| no serious concern | x |
| serious concern | -1 |
| very serious concern | -2 |
| **Rating of evidence** | **In points** |
| High | 4 |
| Moderate | 3 |
| Low | 2 |
| Very Low | 1 |
| **Overall Risk of Bias - 5 domains RoB Tool** | |
| Low | all domains with low risk |
| some concern | unclear at least one domain |
| High | at least one domain with high risk |
