# Supplementary figures and images for: A systematic review and meta-analysis of yoga for arterial hypertension
Source: PLoS One. 2025 May 14;20(5):e0323268. doi: 10.1371/journal.pone.0323268 (PMC12077774; doi:10.1371/journal.pone.0323268)

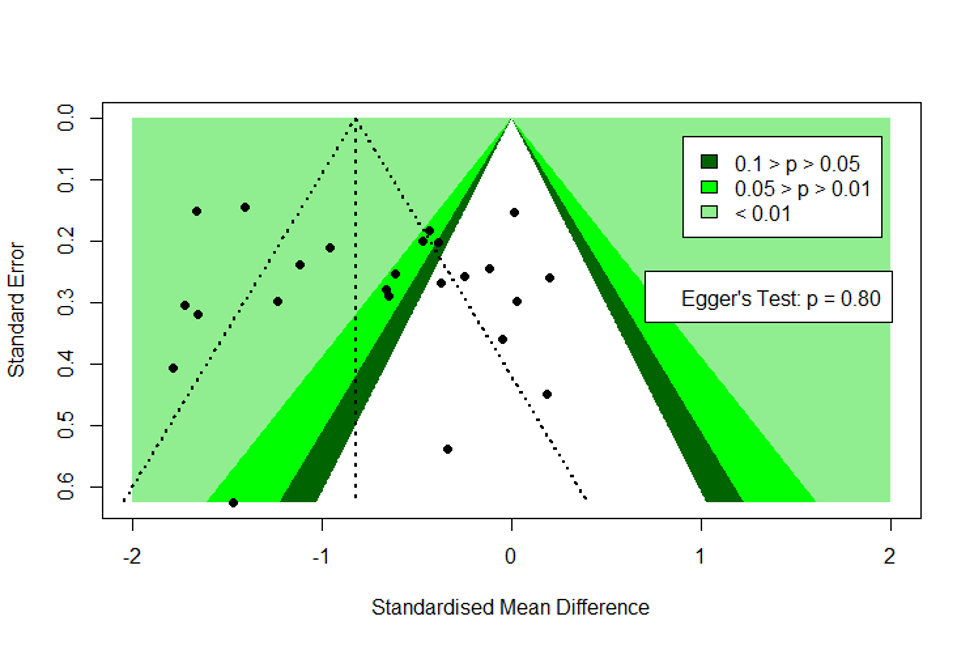

Supplement: S1 Fig — (TIF) [file pone.0323268.s010.tif]

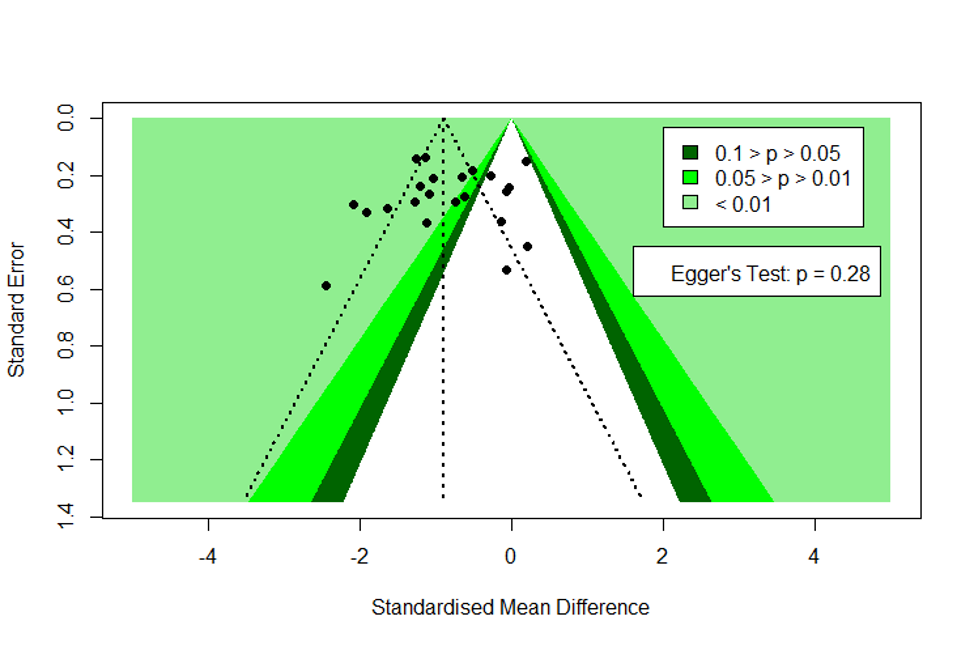

Supplement: S2 Fig — (TIF) [file pone.0323268.s011.tif]

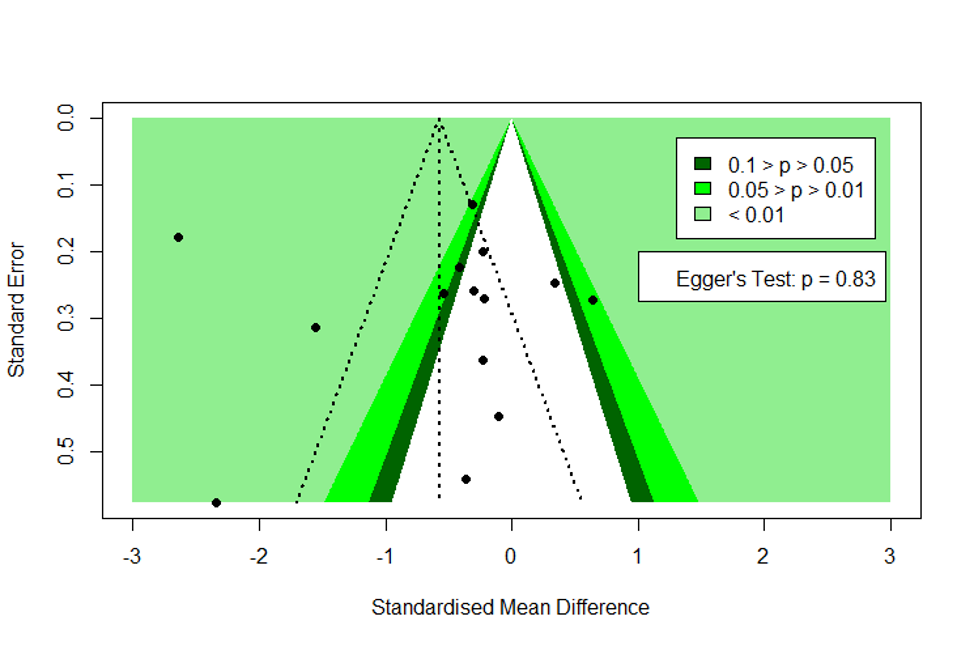

Supplement: S3 Fig — (TIF) [file pone.0323268.s012.tif]

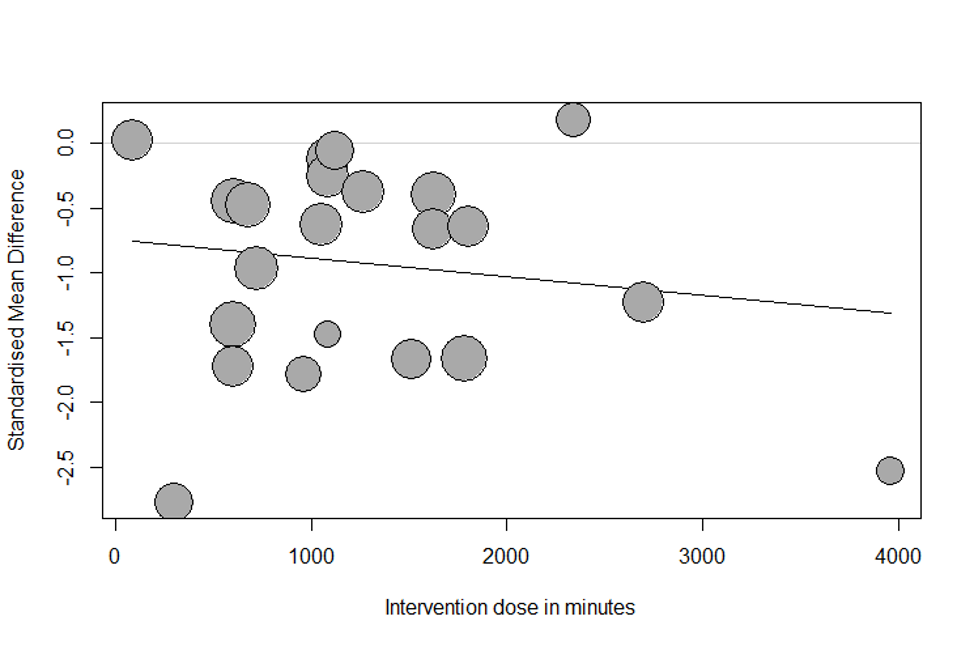

Supplement: S4 Fig — (TIF) [file pone.0323268.s013.tif]

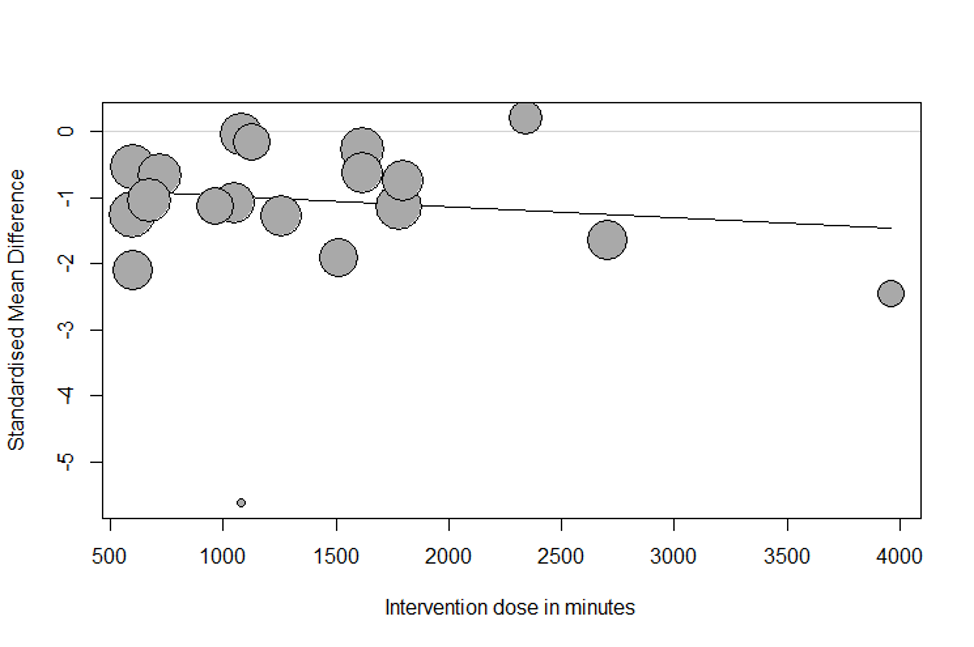

Supplement: S5 Fig — (TIF) [file pone.0323268.s014.tif]

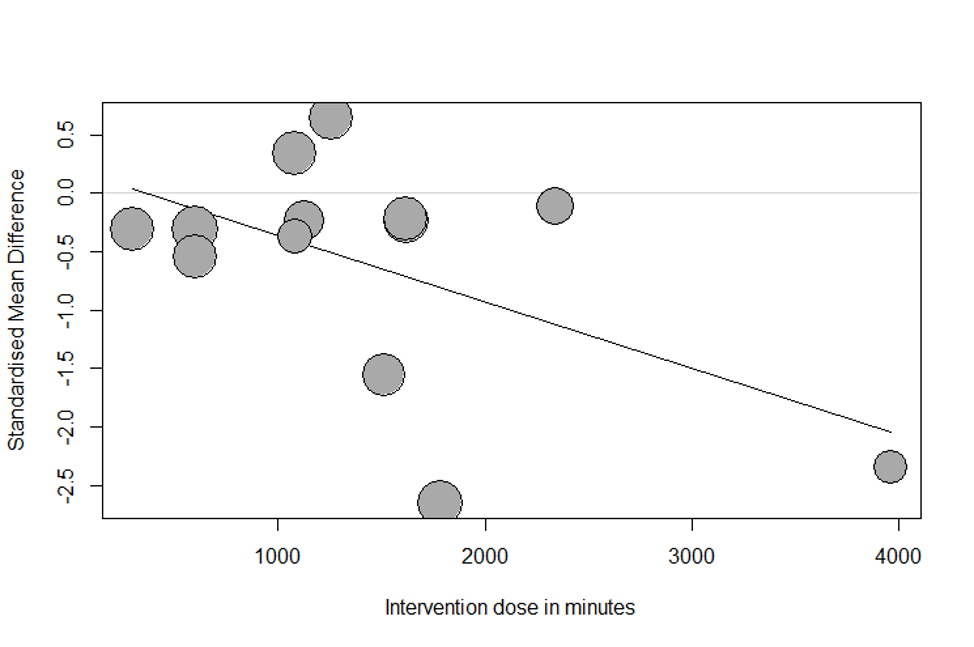

Supplement: S6 Fig — (TIF) [file pone.0323268.s015.tif]
