# Supplementary material for: A systematic review and meta-analysis of yoga for arterial hypertension
Source: PLoS One. 2025 May 14;20(5):e0323268. doi: 10.1371/journal.pone.0323268 (PMC12077774; doi:10.1371/journal.pone.0323268)
Supplement: Attachment — Submitted filename: Rebuttal Letter.docx [file pone.0323268.s017.docx]

Rebuttal Letter

Dear editors and reviewers,

thank you very much indeed for your efforts while reviewing this manuscript and your helpful comments we addressed accordingly. Changes in the manuscript are highlighted in grey as required.

**Editors**

1. Please ensure that your manuscript meets PLOS ONE's style requirements, including those for file naming. The PLOS ONE style templates can be found at

<https://journals.plos.org/plosone/s/file?id=wjVg/PLOSOne_formatting_sample_main_body.pdf> and

<https://journals.plos.org/plosone/s/file?id=ba62/PLOSOne_formatting_sample_title_authors_affiliations.pdf>

Thank you, we did our best and double-checked that our manuscript now meets all PLOS ONE´s style requirements.

2. Thank you for stating the following financial disclosure:

“Wiebke Kohl-Heckl is a member of a post-doc scholarship by the Karl und Veronica Carstens Foundation Germany. The work itself did not receive any specific funding”

Please state what role the funders took in the study.  If the funders had no role, please state: "The funders had no role in study design, data collection and analysis, decision to publish, or preparation of the manuscript."

If this statement is not correct you must amend it as needed.

Please include this amended Role of Funder statement in your cover letter; we will change the online submission form on your behalf.

We included the amended Role of Funder statement in both our cover letter and the online submission.

3. PLOS requires an ORCID iD for the corresponding author in Editorial Manager on papers submitted after December 6th, 2016. Please ensure that you have an ORCID iD and that it is validated in Editorial Manager. To do this, go to ‘Update my Information’ (in the upper left-hand corner of the main menu), and click on the Fetch/Validate link next to the ORCID field. This will take you to the ORCID site and allow you to create a new iD or authenticate a pre-existing iD in Editorial Manager.

Our corresponding author now has an ORCID iD in the updated information.- Https://orcid.org/0009-0001-3049-5089.

4. We notice that your supplementary tables are included in the manuscript file. Please remove them and upload them with the file type 'Supporting Information'. Please ensure that each Supporting Information file has a legend listed in the manuscript after the references list.

We now uploaded all supplementary tables in a file type ‘Supporting information’.

5. Please remove your figures from within your manuscript file, leaving only the individual TIFF/EPS image files, uploaded separately. These will be automatically included in the reviewers’ PDF.

We removed all figures from the manuscript file as needed.

6. We notice that your supplementary figures are uploaded with the file type 'Figure'. Please amend the file type to 'Supporting Information'. Please ensure that each Supporting Information file has a legend listed in the manuscript after the references list.

We changed the file type to *'Supporting Information'* and included a legend in the manuscript.

7. As required by our policy on Data Availability, please ensure your manuscript or supplementary information includes the following:

A numbered table of all studies identified in the literature search, including those that were excluded from the analyses.

For every excluded study, the table should list the reason(s) for exclusion.

If any of the included studies are unpublished, include a link (URL) to the primary source or detailed information about how the content can be accessed.

In the Supporting Information, you can find a table listing all excluded studies and their reason for exclusion, named Supplementary Table S1: Table of excluded studies. All included studies are listed in the table for data extraction Table 1: Study characteristics of eligible studies

A table of all data extracted from the primary research sources for the systematic review and/or meta-analysis. The table must include the following information for each study:

Name of data extractors and date of data extraction

Confirmation that the study was eligible to be included in the review.

All data extracted from each study for the reported systematic review and/or meta-analysis that would be needed to replicate your analyses.

If data or supporting information were obtained from another source (e.g. correspondence with the author of the original research article), please provide the source of data and dates on which the data/information were obtained by your research group.

If applicable for your analysis, a table showing the completed risk of bias and quality/certainty assessments for each study or outcome.  Please ensure this is provided for each domain or parameter assessed. For example, if you used the Cochrane risk-of-bias tool for randomized trials, provide answers to each of the signalling questions for each study. If you used GRADE to assess certainty of evidence, provide judgements about each of the quality of evidence factor. This should be provided for each outcome.

An explanation of how missing data were handled.

This information can be included in the main text, supplementary information, or relevant data repository. Please note that providing these underlying data is a requirement for publication in this journal, and if these data are not provided your manuscript might be rejected.

We have added both a table for the Risk of Bias Assessment and Quality of Evidence Assessment/Results to our Supporting information file (S6 Table, S7 Table and S8 Table).

Table 1 includes all information drawn for the systematic review and meta-analysis. We assessed all included studies for completeness of data relevant to our analysis. This involved reviewing reported outcomes, sample sizes, and any additional variables required for effect size computation. If certain variables critical for analysis or meta-regression were unavailable, we excluded these variables from that specific analysis. To evaluate the potential impact of missing data on our overall findings, we conducted sensitivity analyses. No substantial differences were found, suggesting that missing data did not significantly bias the result.

We also added a further note to the manuscript regarding the needed information:

“All extracted data are presented in Table 1, which only includes studies that were found eligible, thus provides transparency about missing data. All data were extracted by two authors, Christoph Geiger and Wiebke Kohl-Heckl, between April 5^th^, 2024 and May 1^st^, 2024. No further sources but the original articles and their supporting information were used to extract the data needed for the systematic review and meta-analysis. “

**Reviewer #2**

*Overall, the authors have conducted a comprehensive and well-executed systematic review and meta-analysis. The study's strengths include a thorough search strategy, clear inclusion/exclusion criteria, and a robust assessment of risk of bias. The findings are generally consistent with previous research and provide valuable insights into the potential benefits of yoga for managing prehypertension and hypertension.*

Thank you very much for this evaluation of our work! We appreciate it.

*Specific Comments:
Heterogeneity: The authors acknowledge the high degree of heterogeneity among the included studies. It would be beneficial to explore potential sources of heterogeneity, such as differences in yoga styles, duration of interventions, and participant characteristics. This could help identify subgroups that may respond differently to yoga.*

Thank you for this comment. While we have already included this in our limitations section in the original manuscript as it effects heterogeneity and quality of evidence:

“General limitations are based on methodological reasons, incomplete reporting and a low quality of study performance in general. These limitations include different BP measurement techniques, a wide range of BP levels, variation in (control) interventions e.g. passive vs. active control, type of yoga, duration of the intervention period, minutes of yoga applied, as well as medication status, characteristics of study populations and more. “

Further, we also added this to the discussion:

„This heterogeneity of our included studies is based on multiple influencing factors all remaining on study design. As summarized in Table 1, some RCTs based their interventions on breathing exercises or physical exercises only, while others included multiple aspects of Yoga. As well, studies showed various durations and only some collected follow-up data. Further, gender and age distribution varied.“

*Dose-Response Relationship: The authors might consider investigating a dose-response relationship between the intensity or duration of yoga interventions and blood pressure reduction. This could provide valuable information for tailoring yoga programs to individual needs and preferences.*

Thank you for this suggestion. We have already included this aspect in the original manuscript in the meta-regression. As well, it has been mentioned in the discussion:

“We hypothesized that yoga interventions of longer duration would have larger effects on SBP, DBP and HR, unfortunately our meta-regression did not support our assumption. There was no significant correlation between total duration of yoga interventions and lowering effects on SBP, DBP and HR for studies comparing yoga to usual care. […]”
*Quality of Evidence: While the authors note the very low quality of evidence for some of their findings, it's important to discuss the implications of this for clinical practice and future research. Are the observed effects still clinically relevant despite the low quality of evidence? What steps can be taken to improve the quality of future studies?*

Thank you for marking this aspect. We added it to the discussion:

„Even though we only see low-quality evidence so far based on methodological aspects mentioned in the limitations´ section, yoga may play an important role in controlling BP. Yoga has the advantage to meet multiple mechanisms for lowering BP, depending on its incorporated aspects such as relaxation, meditation, breathing and physical activation.”

Further, this has been added to the limitations section:

„To be able to further analyze the role of yoga in controlling BP, future studies need to meet comparable criteria, such as 24h-ABPM, more standardized programs and durations. As well, it would be helpful to integrate long follow-up periods to evaluate long-term effects and duration of such.“

*Long-Term Effects: Given the chronic nature of hypertension, it would be valuable to explore the long-term effects of yoga on blood pressure management. This could include studies with longer follow-up periods to assess the sustainability of the observed benefits.*

Thank you very much for this suggestion. We absolutely see the point and included all available studies that were based on non-single interventions. Unfortunately, there are no studies available that observe longer-term effects as you can extract from the information given in Table 1. The longest follow-up period performed in any of the available studies was 28 weeks.

*Cost-Effectiveness: The authors might consider discussing the cost-effectiveness of yoga as a treatment option for prehypertension and hypertension. This could help inform decision-making by healthcare providers and policymakers.*

Thank you for this great suggestion. We included this in our discussion:

„Especially in prehypertensive individuals, where first-line treatment should be based on lifestyle-modification before including medication, yoga can play an important role. It meets these various aspects of optimizing the autonomous nervous system and includes physical activation, all with the potential of lowering BP. In hypertensive individuals, it can be added as a low-cost therapeutic approach, that can also be performed from home after short period of training or supported by standardized video/online courses, if available. “

*Additional Suggestions:
Subgroup analyses: Consider conducting subgroup analyses based on factors such as age, sex, and baseline blood pressure levels. This could help identify populations that may benefit most from yoga.*

Thank you for these thoughtful suggestions for subgroup analyses. Based on the available information of each study included, you can see that the suggested effects are not available as we do not have studies that are only based on e.g., either male or female groups/certain age groups/etc. In our original manuscript, we have performed subgroup analyses, one that compares prehypertensive to hypertensive to mixed groups. Further evaluation is not possible based on the information given by the original studies

*Mechanism of Action: Discuss potential mechanisms through which yoga may lower blood pressure, such as stress reduction, improved cardiovascular fitness, or alterations in the autonomic nervous system.*

Thank you very much for this idea. We have added this part to the discussion:

„The mechanisms affecting the BP are various. On the one hand it has stress-reducing effects that are most likely due to a regulation of the function of the autonomic nervous system [13]. Besides, the physical postures, depending on the intensity and duration of hold, may result in an optimized cardiac function and muscular function, if executed regularly. This may lead to an overall improved physical function, facilitating daily life.“

*Comparison with Other Lifestyle Interventions: Compare the effects of yoga with other lifestyle interventions, such as exercise, dietary changes, and stress management techniques.*

Thank you for this suggestion. As subgroup analysis, we compared active interventions to usual care/control group. Comparing Yoga to other lifestyle interventions would be a very good idea for a future network-meta-analyses, but defeats the idea of this systematic review and meta-analysis.

*Patient Preferences: Explore patient preferences for yoga as a treatment option. This could help identify factors that may influence adherence and long-term outcomes.*

Thank you for this idea. Unfortunately, neither of the studies mentioned any patient preferences. As this is a very good idea this is a good idea for further research, such as a qualitative study based on patients` interviews, we have added this to our discussion section.

“As well, it is important to evaluate which population may be interested in incorporating yoga into a daily lifestyle for lowering blood pressure. This could for example be achieved by qualitative studies such as patient-focused interviews.”
