# Supplementary material for: A systematic review and meta-analysis of yoga for arterial hypertension
Source: PLoS One. 2025 May 14;20(5):e0323268. doi: 10.1371/journal.pone.0323268 (PMC12077774; doi:10.1371/journal.pone.0323268)
Supplement: Attachment — Submitted filename: Rebuttal Letter Minor Revision.docx [file pone.0323268.s018.docx]

Rebuttal Letter

Dear editors and reviewers,

thank you very much indeed for your efforts while reviewing this manuscript and your helpful comments we addressed accordingly

**Reviewer #2**

*The responses to the given comments and the manuscript have been revised satisfactorily. The revisions appropriately address all the provided comments.
Herewith, I approve the manuscript for further processing.*

Thank you very much for this evaluation of our work! We appreciate it.

**Reviewer #3**

*It is an excellent review with rigorous approach. However, why only three databases were selected? Other relevant database like EBSCO, AYUSH research portal, Web of Science were not used for article search. An author's justification may be added.*

Thank you very much for this comment. As mentioned in our manuscript, our research approach is based on the Preferred Reporting Items for Systematic Reviews and Meta-Analyses (PRISMA) guidelines and the Cochrane recommendations for systematic reviews. Cochrane therefore gives this specific recommendation to utilize three databases:

“The Cochrane Central Register of Controlled Trials (CENTRAL) and MEDLINE, together with Embase (if access to Embase is available to the review team), should be searched for all Cochrane Reviews.” (<https://training.cochrane.org/handbook/current/chapter-04>)

While we decided to utilize Scopus instead of Embase – which was not available to our research team –, Scopus covers about most of the result that can be found via Embase. Scopus additionally covers a broader range of other research fields and met more or our targeted parameters.

We added this recommendation to our manuscript:

“We performed a systematic literature research through the electronic databases Medline/PubMed, Scopus and the Cochrane Central Register of Controlled Trials (CENTRAL), as recommended by Cochrane [29], from their inception until April 5^th^ 2024.”
